# Generalist and Specialist Mite Herbivores Induce Similar Defense Responses in Maize and Barley but Differ in Susceptibility to Benzoxazinoids

**DOI:** 10.3389/fpls.2018.01222

**Published:** 2018-08-21

**Authors:** Huyen Bui, Robert Greenhalgh, Alice Ruckert, Gunbharpur S. Gill, Sarah Lee, Ricardo A. Ramirez, Richard M. Clark

**Affiliations:** ^1^School of Biological Sciences, University of Utah, Salt Lake City, UT, United States; ^2^Department of Biology, Utah State University, Logan, UT, United States; ^3^Center for Cell and Genome Science, University of Utah, Salt Lake City, UT, United States

**Keywords:** Maize (*Zea mays* L.), *Hordeum vulgare*, *Tetranychus urticae*, *Oligonychus pratensis*, benzoxazinoid, spider mite, herbivore, HDMBOA

## Abstract

While substantial progress has been made in understanding defense responses of cereals to insect herbivores, comparatively little is known about responses to feeding by spider mites. Nevertheless, several spider mite species, including the generalist *Tetranychus urticae* and the grass specialist *Oligonychus pratensis*, cause damage on cereals such as maize and wheat, especially during drought stress. To understand defense responses of cereals to spider mites, we characterized the transcriptomic responses of maize and barley to herbivory by both mite species, and included a wounding control against which modulation of defenses could be tested. *T. urticae* and *O. pratensis* induced highly correlated changes in gene expression on both maize and barley. Within 2 h, hundreds of genes were upregulated, and thousands of genes were up- or downregulated after 24 h. In general, expression changes were similar to those induced by wounding, including for genes associated with jasmonic acid biosynthesis and signaling. Many genes encoding proteins involved in direct defenses, or those required for herbivore-induced plant volatiles, were strongly upregulated in response to mite herbivory. Further, biosynthesis genes for benzoxazinoids, which are specialized compounds of Poaceae with known roles in deterring insect herbivores, were induced in maize. Compared to chewing insects, spider mites are cell content feeders and cause grossly different patterns of tissue damage. Nonetheless, the gene expression responses of maize to both mite herbivores, including for phytohormone signaling pathways and for the synthesis of the benzoxazinoid 2-hydroxy-4,7-dimethoxy-1,4-benzoxazin-3-one glucoside, a known defensive metabolite against caterpillars, resembled those reported for a generalist chewing insect, *Spodoptera exigua*. On maize plants harboring mutations in several benzoxazinoid biosynthesis genes, *T. urticae* performance dramatically increased compared to wild-type plants. In contrast, no difference in performance was observed between mutant and wild-type plants for the specialist *O. pratensis*. Collectively, our data provide little evidence that maize and barley defense responses differentiate herbivory between *T. urticae* and *O. pratensis*. Further, our work suggests that the likely route to specialization for *O. pratensis* involved the evolution of a robust mechanism to cope with the benzoxazinoid defenses of its cereal hosts.

## Introduction

Cereal crops of the grass family (Poaceae) account for the majority of human calories, and reductions in their yield dramatically impact human welfare. Abiotic factors, such as drought, are a major source of unrealized yield (Boyer, 1982), while another well-characterized source of loss is from herbivory by insects (Oerke, 2006). Spider mites (Acari: Tetranychidae) belong to the Chelicerata, an arthropod lineage that diverged more than 450 million years ago (Dunlop, 2010), and hence evolved herbivory independently from insects. Crops including maize (*Zea mays*) and wheat (*Triticum* sp.) are susceptible not only to insects but also to spider mites, especially during drought conditions (Al-Kaisi et al., 2013), where yield losses as high as 47.2% for maize have been reported (Bacon et al., 1962). Nevertheless, relatively little is known about the molecular nature of the defenses plants use to deter spider mites, especially for grasses.

As shown by molecular studies of plant–herbivore interactions, largely with insects and dicots such as *Arabidopsis thaliana* and tomato (*Solanum lycopersicum*), many plants complement constitutive defenses (like trichomes) with rapid, inducible ones that negatively impact herbivores (Howe and Jander, 2008). For instance, herbivore-associated triggers like physical damage, oral secretions, or frass, alone or in combination, lead to changes in the production of specialized metabolites or defensive proteins that deter herbivores (Howe and Jander, 2008; Ray et al., 2015). In dicots, molecular responses to insect herbivores are mediated largely by phytohormones, especially jasmonates (jasmonic acid, or JA, and its derivatives or conjugates), which induce transcriptomic reprogramming within hours (Howe and Jander, 2008). Some defenses act directly, such as toxic compounds or protease inhibitors that retard digestion in an herbivore’s gut. Others act indirectly, like plant volatiles, which can serve as olfactory cues for predators to locate herbivores at feeding sites (Turlings and Erb, 2018).

The type and magnitude of inducible defenses is influenced by several factors. One of these is feeding guild. Chewing insects like caterpillars, for instance, cause extensive tissue damage and elicit different defense responses compared to phloem-feeding insects like aphids, which cause minimal loss of plant tissue (Howe and Jander, 2008). Additionally, plant responses to generalist herbivores, to which ∼10% of plant-feeding insects belong (Ali and Agrawal, 2012), can differ from those induced by specialists. Generalist herbivores feed on hosts in many plant families. They are typically thought to rely on broad detoxification capabilities to overcome the challenges they encounter on phylogenetically (and chemically) divergent plant hosts (Dermauw et al., 2013), or to potentially suppress plant defense responses that are broadly conserved (Ali and Agrawal, 2012). Alternatively, some specialists have evolved the ability to suppress or otherwise circumvent plant defenses, potentially ameliorating the role of detoxification, or instead have evolved highly specialized detoxification abilities to cope with the toxins they encounter in their preferred plant hosts (Dobler et al., 2012; Glas et al., 2014; Maag et al., 2014; Wouters et al., 2014).

Like dicots, monocots, including grasses, are attacked by generalist and specialist herbivores of diverse feeding guilds, including leaf-chewing (e.g., caterpillars) and piercing-sucking (e.g., aphids and whiteflies). As for dicots, JA signaling and the production of specialized compounds feature prominently in monocot responses to insect herbivory (Meihls et al., 2012; Tzin et al., 2015a, 2017). Of the downstream specialized compounds in grasses, the best studied are benzoxazinoids, which are 1,4-benzoxazin-3-one derivatives produced by cereals including maize, wheat, and rye (Zúñiga et al., 1983; Niemeyer, 2009). In maize, levels of benzoxazinoids are highest in seedlings (Cambier et al., 2000), but can be locally induced at feeding sites in the leaves of older plants (Köhler et al., 2015; Maag et al., 2016). The most studied benzoxazinoid, 4-dihydroxy-7-methoxy-1,4-benzoxazin-3-one (DIMBOA), is stored in vacuoles as an inactive glucoside (Glc) conjugate. Upon tissue damage by herbivores, DIMBOA-Glc, as well as derivatives such as 2-hydroxy-4,7-dimethoxy-1,4-benzoxazin-3-one glucoside (HDMBOA-Glc), are exposed to glucosidases in plastids (Meihls et al., 2012). This leads to the release of the aglucones, which are toxic to herbivores, potentially by several modes of action (Wouters et al., 2016).

**FIGURE 1:**
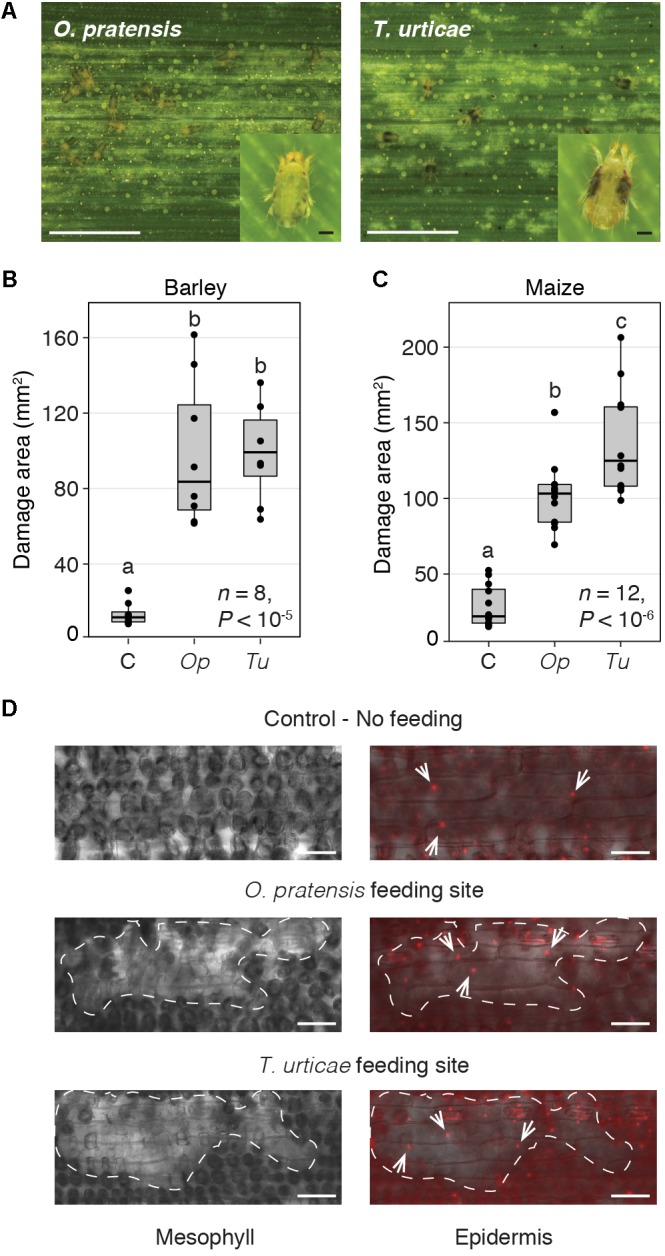
*O. pratensis* and *T. urticae* morphology, tissue damage, and feeding strategy. **(A)** *O. pratensis* and *T. urticae* feeding causes light colored (chlorotic) spotting on barley leaves, as well as on maize (see also **Supplementary Figure 2**) (scale bars: 2.5 mm). Insets show adult *O. pratensis* and *T. urticae* females (scale bars: 100 μm). **(B,C)** Total area of damage on barley and maize leaf enclosures, respectively, for uninfested leaves (C, control), and those subjected to *O. pratensis* (*Op*) and *T. urticae* (*Tu*) feeding at 24 h. *P*-values are from an ANOVA, and letters indicate significant differences between comparisons (*P* < 0.05, Tukey’s HSD test; *n*: number of biological replicates). In the boxplots, circles represent individual data points. **(D)** Leaf cell damage caused by *O. pratensis* and *T. urticae* feeding on barley. Clusters of empty mesophyll cells at mite feeding sites (left) are denoted by dashed lines; a focal plane through the epidermis is shown at right with nuclei visualized by propidium iodide staining (white arrows). Scale bar: 100 μm.

Several spider mite species are significant field pests on cereals. These include *Tetranychus urticae* (the two-spotted spider mite) on maize, and *Oligonychus pratensis* (the Banks grass mite) on both maize and distant relatives including wheat (**Figure 1A**; Brandenburg and Kennedy, 1982; Mansour and Bar-Zur, 1992; Archer and Bynum, 1993; Tadmor et al., 1999; Blasi et al., 2015). *T. urticae* is an extreme generalist that has been documented on more than 100 plant families (Grbić et al., 2011). In contrast, *O. pratensis* is a specialist on plants in the Poaceae, though it has been reported on a few non-grass hosts including date palm (which is also a monocot) (Ward et al., 1972; Foster et al., 1977; Chandler et al., 1979; Holtzer et al., 1984; Archer and Bynum, 1993; Bynum et al., 2015; Negm et al., 2015). As cell-content feeders, spider mites belong to a different feeding guild than the best studied insect herbivores (Bensoussan et al., 2016). Currently, knowledge of plant responses to spider mites comes mainly from *A. thaliana*, tomato, and grapevine (*Vitis vinifera*), where *T. urticae* feeding was shown to induce robust JA responses (Zhurov et al., 2014; Martel et al., 2015; Díaz-Riquelme et al., 2016). This suggests that *T. urticae* relies in large part on a broad detoxification capacity to enable its exceptionally wide host range. Nonetheless, the sister species *T. evansi*, a specialist of plants in the Solanaceae, has been documented to suppress plant defenses in tomato (Alba et al., 2015). Further, another specialist mite herbivore of tomato, *Aculops lycopersici*, is able to potently suppress plant defenses (Glas et al., 2014). While *O. pratensis* is a pest on diverse cereal crops like maize and wheat, little is known about its ancestral host range within Poaceae. Whether the grass specialist *O. pratensis* relies on detoxification, suppression of plant defenses, or both, to colonize its Poaceae hosts is unknown.

In this study, we have characterized defense responses to herbivory by *O. pratensis* and *T. urticae* in grasses. As a plant host, we chose maize, for which defense responses to insects have been comparatively well studied (Meihls et al., 2012; Tzin et al., 2015a, 2017). We also included barley (*Hordeum vulgare*) as it is a close relative of wheat with a more tractable genome (Mayer et al., 2012). By including both maize and barley, we were able to compare variation in plant defense responses to both mite species in each of two major cereal crops in phylogenetically distant lineages within Poaceae (subfamilies Panicoideae and Pooideae, respectively).

## Materials and Methods

### Biological Materials and Maintenance of Stocks

Seeds for barley (cultivar Morex) were kindly provided by A. Fischer (Montana State University, Bozeman, MT, United States), while those for maize inbred B73 were kindly provided by G. Drews (University of Utah, Salt Lake City, UT, United States). Seed stocks for maize inbred W22, as well as homozygous lines for previously characterized *Ds* insertions in *BX1*, *BX2*, and *BX6* on the W22 background (Tzin et al., 2015a), were kindly provided by G. Jander (Boyce Thompson Institute, Ithaca, NY, United States). For the study, a *T. urticae* colony (strain W-GR) was established from *T. urticae* collected from a community garden and from a greenhouse site in Salt Lake City, UT, United States. Prior to this study, the W-GR strain was maintained on whole kidney bean plants (*Phaseolus vulgaris*) for more than 30 generations using established rearing methods (Van Leeuwen et al., 2012). Before being used as a source of mites for plant infestation experiments (see below), mites of W-GR were acclimated on barley (Morex) or maize (B73) for at least two generations to remove possible maternal effects on host use that might impact plant responses. For propagation, 8–10 week old barley and maize plants were used to maintain bulk populations of at least several thousand mites. For *O. pratensis*, a field-collected strain was acquired from maize (Logan, UT, United States); propagation of the *O. pratensis* strain, and acclimation prior to the respective experiments on barley and maize, was as for *T. urticae* except that *O. pratensis* was never maintained on kidney beans, which are not a host.

For maintaining mite colonies, barley and maize plants were germinated and grown in Metro-Mix^®^ 900 growing mix (Sun Gro^®^, Agawam, MA, United States) and watered from below as needed (pots were placed in trays to which water was added). Barley plants were germinated and grown in a walk-in growth chamber with a 16-h light/8-h dark photoperiod with 170–200 μmol m^-2^ s^-1^ light at 20°C and 60% humidity. Maize plants were germinated and propagated in a greenhouse with a 16-h light/8-h dark photoperiod at an approximate temperature of 25°C. Maize and barley plants were fertilized weekly with 200 ppm NutriCulture Cal-Mag Special 16N-3P-16K (for maize) and NutriCulture Mag-Iron Special 18N-6P-18K (for barley) (Plant Marvel Laboratories, Chicago Heights, IL, United States). At the respective leaf stage, plants were moved to an isolated room maintained at room temperature (∼22°C) for mite propagation, with new plants placed next to and touching previously infested plants to allow mites to move to fresh hosts. Mites from these colonies were used for all experiments unless otherwise noted.

### Quantification of Feeding Damage

To assess the extent of foliar damage caused by mite feeding, mites of each species were placed on either the fifth leaves of 30-day-old barley plants or on the fourth leaves of 22-day-old maize plants. The studies for barley and maize were performed in a walk-in growth chamber or a greenhouse, respectively, under the conditions described above. Briefly, barley and maize seeds were sown 1–2 cm deep in 5 × 5 cm plastic pots in Metro-Mix^®^ 900 growing mix. Ten days after sowing, seedlings were transplanted into 20 cm diameter pots. As the maize plants were grown in a greenhouse bay, plants were maintained inside insect-free enclosures until the day before the experiment to exclude potential greenhouse pests (insect enclosures were constructed with PVC piping and No-See-Um nylon netting, BioQuip Products, Compton, CA, United States, catalog number 7250NSB). To minimize the impact of environmental variation, a randomized block design was employed.

While mites cannot fly, they can disperse quickly by crawling on leaves. Therefore, for both control plants and those used for mite infestations, we established leaf enclosures using non-phytotoxic wax barriers that mites do not cross, an approach previously employed in studies with both mite species (Feese and Wilde, 1977; Mansour and Bar-Zur, 1992). For our study, we used Tree Tanglefoot wax (The Scotts Miracle-Gro Company, Marysville, OH, United States), with two barriers established perpendicular to the length of the leaf blades (Tanglefoot was placed in stripes across the adaxial and abaxial surfaces of leaves and joined at the edges; **Supplementary Figure 1**). Mites added between the two barriers were thus free to move along and between the leaf surfaces internal to the Tanglefoot boundaries. Maize leaves are well separated, and hence enclosures were easily established on free-standing plants. However, the plant architecture of barley presented an additional challenge, necessitating immobilization of leaf-harboring enclosures as shown in **Supplementary Figure 1**.

Twenty-four hours after establishing 12.5 cm Tanglefoot enclosures, 250 and 350 female mites were used to infest barley and maize leaves, respectively; as maize leaves are wider, more mites were added to keep mite density roughly constant (no mites were added to enclosures on the control plants). Adult mites of both species, which are only ∼0.6 mm in length (**Figure 1A**, insets), were collected under dissection microscopes from the bulk populations maintained on the respective hosts. To do this, defined numbers of adult females were harvested into 200 μL barrier pipet tips by suction (the large ends of pipet tips were attached to vacuum lines). At the time of collection, tips with mites were immediately placed onto ice. On ice, mites are less physically active and less likely to spin webs, which can otherwise lead to complications for subsequent release. To release mites onto leaf enclosures, pipet tips with mites were removed from ice, the tops of the tips were cut off with scissors (to increase the bore size for release), and mites were gently poured onto the leaf surface along the length of the enclosures.

At 24 h post-infestation, leaf enclosures were harvested by cutting across the leaf blade immediately internal to the Tanglefoot barriers. Both sides of each leaf segment were then scanned at 1200 dpi using an Epson Perfection V550 Photo scanner (Epson, Suwa, Japan). To ensure the mite damage was quantified from leaf segments of exactly the same length, the leaf segments were immobilized to a frame to reveal the middle 7.5 and 10 cm for barley and maize enclosures, respectively, before scanning. Barley leaf segments were sufficiently flat to be scanned without additional manipulation. In contrast, maize leaf segments could not be scanned directly due to pronounced midribs; therefore, leaf segments harvested from the enclosures were cut along the midrib, and the midrib and two halves of the leaf blade were pressed flat for scanning (**Supplementary Figure 2**). The resulting images were processed using the thresholding function of the FIJI software (Schindelin et al., 2012), which allowed selection of light colored spots caused by mite feeding. The total area of damage on the adaxial and abaxial leaf surfaces was then quantified. Statistical analyses of leaf feeding damage were performed with analysis of variance (ANOVA) in R version 3.3.2 (R Core Team, 2016), with pairwise comparisons among treatments subsequently performed with Tukey’s HSD test.

### Cell Imaging at Mite Feeding Sites

Barley leaves were infested with *T. urticae* and *O. pratensis* for 24 h as described for quantification of mite feeding damage. Uninfested leaves were used as controls. Barley leaf segments with and without mite feeding were dehydrated through an ethanol series (15, 50, 75, and 95%) for 30 min each under vacuum, then decolorized in 200 proof ethanol overnight at 4°C. Decolorized leaf segments were rinsed twice in water, then incubated in 1 μg/mL propidium iodide for an hour. Stained leaf segments were then rinsed and mounted in water for imaging, and Z-series images were collected at 2 μm intervals through the epidermis and mesophyll layers of the leaf specimens using a Zeiss Axio Observer Z1 microscope (Zeiss, Baden-Württemberg, Germany). Fluorescence signal from propidium iodide was detected using an excitation filter of 559–585 nm and an emission filter of 600–690 nm. Images were processed using FIJI (Schindelin et al., 2012), Adobe Photoshop CC 2017, and Adobe Illustrator CC 2017 (Adobe, San Jose, CA, United States).

### Collection of Plant Tissue for Transcriptomic Studies

Plant leaf tissue was collected from barley (Morex) and maize (B73) plants with the following treatments: (1) control (uninfested leaves), (2) wounding, (3) *T. urticae* infestation, and (4) *O. pratensis* infestation. As a time course, we selected 2- and 24-h time points because dynamic expression changes in defense genes in response to *T. urticae* were observed in the dicots *A. thaliana*, tomato, and grapevine within 24 h (Zhurov et al., 2014; Martel et al., 2015; Díaz-Riquelme et al., 2016). Further, in maize, time points for analogous studies with insects from several feeding guilds were similar, and included 24-h time points (Tzin et al., 2015a, 2017). The experimental design employed for assessing transcriptomic responses was similar to that for assessing quantification of feeding damage with several modifications. For barley, 30-day-old plants were used with enclosures of length 10 cm on the fifth leaves, and for maize, 22-day-old plants were used with enclosures of length 9 cm on the fourth leaves (mite density was therefore held roughly constant). A randomized block design was used to minimize environmental variation. For the wounding treatments, plant tissue in enclosures was gently pressed between 2 pieces of 60 grit sandpaper (particle size ∼260 μm; the blades on each side of the midrib were wounded along the lengths of the enclosed leaf segments). For mite infestation, 400 and 200 adult female mites were used per leaf enclosure for the 2- and 24-h time points, respectively. Briefly, because it takes tens of minutes to several hours for released mites to fully untangle themselves, disperse, and start feeding, the density of applied mites was doubled for the 2-h time point to ensure feeding by 2 h. For RNA preparation, we collected tissue from within the enclosures as a major component of induced plant defense responses is local (Maag et al., 2016; Tzin et al., 2017). Following best practices in the field (Tzin et al., 2015a, 2017), mite infestation and wounding treatments were staggered prior to a single collection time (**Supplementary Figure 3**). In all cases, a biological replicate consisted of the entire leaf enclosure from a single leaf. To minimize the effects of the circadian cycle, for both the barley and maize experiments all samples were collected within a 15-min time window 3 h into the 16-h light period.

### RNA Isolation, RNA-Seq Library Construction, and Sequencing

Total RNA was prepared from leaf material from Tanglefoot enclosures with the DirectZol RNA extraction kit (Zymo Research, Irvine, CA, United States). Barcoded RNA-seq libraries were constructed at the High-Throughput Genomics Core Facility (University of Utah) using the Illumina (Illumina, San Diego, CA, United States) TruSeq Stranded mRNA Library Preparation Kit with poly(A) selection, and 125 bp paired-end reads were generated on an Illumina HiSeq 2500 sequencer with HiSeq SBS Kit v4 sequencing reagents. Briefly, four lanes were run in total for each of the barley and maize experiments, each consisting of 28 samples (four replicates each for the control, wounding at 2 and 24 h, *T. urticae* infestation at 2 and 24 h, and *O. pratensis* infestation at 2 and 24 h). Biological replicates were evenly distributed across the four sequencing lanes to reduce the possibility of confounding due to lane-level effects.

### Detection of Differentially Expressed Genes

The barley genome version ASM3268v1 (Release 33) (Mayer et al., 2012) and the maize genome version AGPv3 (Release 31) (Schnable et al., 2009) were downloaded from Ensembl (Yates et al., 2016). To examine expression of benzoxazinoid biosynthesis genes in maize, the gene model for *BX7* (*GRMZM2G441753*) was taken from an earlier annotation (Ensembl Release 20), as it was considered a low-confidence model and not included in the newer AGPv3 release. RNA-seq reads were aligned to their respective genomes using the two-pass alignment mode of STAR 2.5.2b (Dobin et al., 2013) with a maximum intron size of 20 kb. The number of reads uniquely aligned to each locus was counted using HTSeq 0.6.0 (Anders et al., 2015) with “--strand reverse” and “--feature transcript”. Differentially expressed genes (DEGs) were detected using the DESeq2 package (version 1.14.0) (Love et al., 2014) with a false discovery rate (FDR) adjusted *P*-value of 0.01 and an absolute value log_2_ fold change cutoff of 1. Fragments per kilobase of transcript per million mapped reads (FPKM) (Mortazavi et al., 2008; Trapnell et al., 2010) values were calculated with Python scripts using the BCBio^1^ GFF parser.

### Cluster Analyses

Independently for each of the maize and barley time course RNA-seq data sets, gene-level *k*-means clustering was performed on the set of genes that was differentially expressed in at least one treatment. Briefly, the log_2_ fold change estimates of each gene across treatments compared to the control were used as the input to the *kmeans* function of the base “stat” package in R with the default Hartigan and Wong algorithm. Different *k* numbers were tested with *k* = 6 chosen as it resulted in clusters with distinct expression profiles across the treatments. The *kmeans* function was run with 100 iterations (nstart = 100) of the algorithm to optimize the clustering output. To compare the expression profiles across host species, the centers of the barley and maize gene *k*-means clusters were subjected to hierarchical clustering using the *hclust* function in R (distance: squared Euclidean; linkage: complete). While the *k*-means clusters were numbered arbitrarily by the *kmeans* function, barley and maize gene clusters with similar expression patterns identified by hierarchical clustering were manually renumbered for simplicity of comparison.

### Gene Ontology Annotations and Gene Set Enrichment Analysis

Gene ontology (GO) annotations of the barley genome version ASM3268v1 (Release 33) and maize genome version AGPv3 (Release 31) were obtained by parsing the respective EMBL flat files provided by Ensembl (Biopython package SeqIO, version 1.69; Cock et al., 2009). The GO annotations were used to classify gene sets using the biological process (BP) and molecular function (MF) ontologies. The MF ontology was used to identify genes encoding defensive proteins and enzymes (i.e., protease inhibitors, chitinases, and terpene synthases).

Gene set enrichment analysis was performed using the BP ontology. To enable a comparison of gene set enrichments between barley and maize gene sets, we modified the BP ontology annotation of each species so that one-to-one orthologs between barley and maize were associated with the same GO terms. The one-to-one orthologs were determined by reciprocal BLASTP (BLAST+ version 2.5.0+; Camacho et al., 2009) searches with an *E*-value cut-off <10^-5^. The search was limited to the longest isoform of each protein. For each gene with a best reciprocal BLASTP hit, the list of BP ontology terms was updated to contain the union of annotated BP ontology terms from the barley and maize orthologs. Gene set enrichment analyses were performed using the “weight01” algorithm with Fisher’s test statistic as implemented in the topGO 2.32.0 package (Alexa et al., 2006).

### Hormonometer Analyses

We adapted the Hormonometer tool (Volodarsky et al., 2009) to assess plant hormone signaling in barley and maize following the approach used by Tzin et al. (2015a, 2017) in studies of maize responses to herbivory by insects. This tool searches for the similarity of gene expression changes to signatures of transcriptomic responses diagnostic for specific plant hormone signaling pathways. Briefly, experimentally assessed reference expression data sets are available from *A. thaliana*, and were generated by exogenous application of plant hormones (or hormone precursors) that induce JA, salicylic acid (SA), ethylene, abscisic acid (ABA), brassinosteroid, cytokinin, auxin, and gibberellic acid (GA) signaling (Volodarsky et al., 2009). As the Hormonometer tool was developed with *A. thaliana* expression data, to use the tool with barley and maize we performed reciprocal BLASTP searches with *A. thaliana* proteins to identify orthologs in both grass species. As a result, we identified 8236 barley genes and 8904 maize genes expressed in our study that had corresponding *Arabidopsis* Probeset IDs (**Supplementary Data Sheets 1**, **2**, respectively), and we used these as input for the Hormonometer tool to assess the similarity of gene sets induced by spider mite feeding and wounding to those induced by specific phytohormones.

### Peroxidase Activity Assays

Plants were grown and infested with mites as described for damage quantification. The leaf samples were collected 24 h after infestation and ground to a fine powder in liquid nitrogen. Peroxidase activity in leaf samples was quantified with a microplate reader (Biotek EPOCH, Winooski, VT, United States) as described previously (Ramirez and Spears, 2014). Briefly, frozen leaf powder was thawed and suspended in 1 mL of sodium phosphate buffer (0.05 M, pH 7.0). Leaf homogenates were centrifuged for 12 min at 12,000 rpm to extract soluble leaf proteins in the supernatant. Peroxidase activity was detected in soluble protein at 470 nm following the oxidation of guaiacol for 1 min (Moran and Cipollini, 1999), and expressed as the change in absorbance per mg of total protein. The impacts of the treatments on peroxidase activity were assessed by ANOVA factoring in the block design; where significant effects were observed, Tukey’s HSD tests were performed.

### Mite Productivity on *bx* Mutant Plants

To assess the impact of mutations in genes in the maize benzoxazinoid pathway on mite infestation, seeds of W22, *bx1::Ds*, *bx2::Ds*, and *bx6::Ds* (Tzin et al., 2015a) were germinated directly in 15 cm diameter pots. At 2 weeks (approximately the three-leaf stage), plants of the four genotypes were arranged in a randomized block design with 16 replicates of each genotype (eight plants per block). Three 1–2-day-old adult females from synchronized mite populations were then added to each plant. To do this, three mites were sucked into barrier pipet tips as described previously, and the mites were tapped to the bottom of the tips against the barriers. The tops of the tips were then cut off to allow mites to escape, and single tips were immediately taped to the bottom of individual plants with the top of the cut tip pointing up and touching the second leaf. Synchronized mite populations were obtained by placing fertilized female mites on detached B73 maize leaves, allowing the females to lay eggs for 2 days, and then removing them (the resulting population of female mites was thus approximately synchronized). To prevent detached leaves from drying out, they were placed on wet cotton and their edges were sealed with Tanglefoot wax. Eighteen days after adding tips with mites to plants, entire plants were collected and kept at 4°C (which arrests mite reproduction and development). The number of eggs and viable mites (larvae, nymphs, and adults) found on each plant was then subsequently counted under a dissecting microscope. The impact of maize genotype on mite productivity by species per plant was assessed with ANOVA factoring in the block design. Subsequent pairwise tests were performed with Tukey’s HSD method.

## Results

### *O. pratensis* and *T. urticae* Cause Similar Patterns of Tissue Damage

To assess responses of barley (cultivar Morex) and maize (inbred B73) to specialist and generalist spider mite herbivores, we examined transcriptomic responses to *O. pratensis* and *T. urticae* feeding at 2 and 24 h. As the magnitude of wounding can impact interpretation of transcriptomic responses, we first assessed the extent of plant tissue damage from both mite species. For *O. pratensis* and *T. urticae*, and for barley and maize, macroscopic damage from mite feeding at 24 h consisted of fine white stippling (**Figure 1A** and **Supplementary Figure 2**). For both plant hosts and for both mite herbivores, significant plant damage was observed compared to uninfested leaves following damage quantification from leaf scans (*P* < 0.05, ANOVA with Tukey’s HSD method to correct for multiple tests; **Figures 1B,C**). At 24 h, areas of stippling caused by *O. pratensis* and *T. urticae* on barley were not significantly different (**Figure 1B**). In contrast, a significant albeit modest increase in damage was observed for maize leaves infested with *T. urticae* relative to those exposed to *O. pratensis* (**Figure 1C**). It should be noted, however, that the sample size, and hence power to detect an effect, was larger for maize; this was a function of performing maize studies in a large greenhouse as opposed to a growth chamber as was used for barley (see the section “Materials and Methods”).

We also examined the microscopic pattern of damage caused by *O. pratensis* and *T. urticae* on barley leaves, for which the even epidermis is straightforward to image with differential interference contrast and fluorescence microscopy in cleared tissue. For both mite species, clusters of mesophyll cells were empty at feeding sites (or minimally, were devoid of chloroplasts that are dark in appearance; **Figure 1D**). The overlying epidermis, in which cells lack chloroplasts, nonetheless appeared to be intact as assessed by the presence of propidium iodide stained nuclei in pavement cells.

### Spider Mite Herbivores Induce Similar Transcriptomic Responses on Barley and Maize

To examine transcriptomic responses to herbivory by *O. pratensis* and *T. urticae*, we collected plant tissue from within leaf enclosures at 2 and 24 h after mite infestation. Quantification of gene expression from alignments of the resultant RNA-seq reads from four biological replicates for controls and treatments revealed that 21,472 of 26,066 (82.4%) high-quality barley genes were expressed (i.e., had non-zero read counts), as were 30,279 of 39,625 (76.4%) high-quality genes in maize (see the section “Materials and Methods”).

**FIGURE 2:**
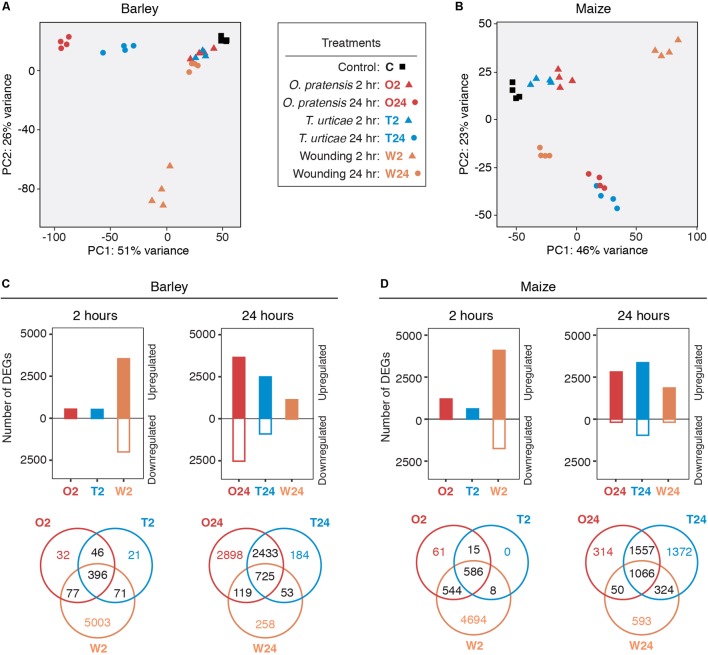
Dynamic gene expression changes in barley and maize in response to mite herbivory and wounding. **(A,B)** PCAs of expression data for control, mite infested, and wounded leaves in barley and maize, respectively. Treatments and time points are as indicated in the legend (center; hour: hr). **(C,D)** Number of significantly up- and downregulated genes detected in each treatment for barley and maize, respectively (FDR-adjusted *P*-value of 0.01, absolute value log_2_ fold change cutoff of 1). Expression differences are broken out by time points (2 and 24 h, left and right for each plant host), and Venn diagrams show the overlap of DEGs among treatments (bottom).

As assessed with a principal component analysis (PCA) using controls and all treatments, biological replicates were tightly clustered (**Figures 2A,B**). For the 2-h time point, replicates for both *O. pratensis* and *T. urticae* clustered nearby but were separate from controls in both barley and maize. A similar but more distinct pattern was observed at 24 h. Although dramatic differences in plant responses to the two mite species were not readily apparent for most contrasts, *O. pratensis* replicates at 24 h clustered farther away from control samples in barley than did those for *T. urticae*, albeit along the same PCA axis.

We used DESeq2 (Love et al., 2014) to detect DEGs – as assessed with a FDR-adjusted *P*-value of 0.01 and an absolute value log_2_ fold change cutoff of 1 – between treatments and controls for barley and maize (**Figures 2C,D**, respectively; results of DEG analyses for all comparisons are given in **Supplementary Data Sheet 3** for barley and **Supplementary Data Sheet 4** for maize). In response to feeding by both *O. pratensis* and *T. urticae*, hundreds and thousands of DEGs were detected in both barley and maize at 2 and 24 h, respectively. In contrast to the 2-h time point, for which nearly all expression changes involved upregulation, both up- and downregulation were observed at 24 h.

**FIGURE 3:**
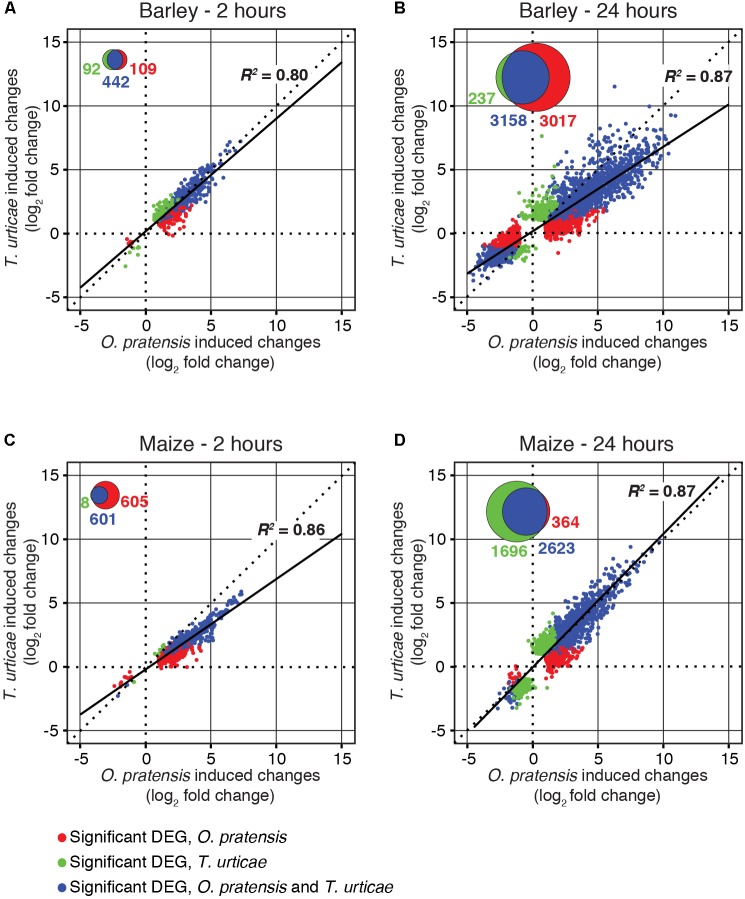
*O. pratensis* and *T. urticae* induce similar changes in gene expression in both barley and maize. Scatter plots of log_2_ fold changes for DEGs (FDR-adjusted *P*-value of 0.01, absolute value log_2_ fold change cutoff of 1) in barley **(A,B)** and maize **(C,D)** in response to *T. urticae* and *O. pratensis* herbivory at two time points (2 h: **A**,**C**; 24 h: **B**,**D**). For inclusion in a given analysis, a gene had to be detected as differentially expressed in response to at least one herbivore (see Venn diagram insets, and legend, bottom left).

The partial overlap of DEGs between mite treatments (**Figures 2C,D**) raised the possibility that components of plant defense responses to the generalist and specialist mites differed. To investigate this further, we generated scatter plots of log_2_ fold changes for genes responding to mite herbivory at the 2- and 24-h time points for combinations of plant and mite species (**Figure 3**). Consistent with the PCAs (**Figures 2A,B**), transcriptomic responses to feeding by *O. pratensis* and *T. urticae* were highly correlated at both time points in each species (*R*^2^-values between 0.8 and 0.9, all *P-*values < 10^-16^; **Figure 3**). Our findings, therefore, revealed no compelling evidence for large-scale, qualitative differences in plant gene expression responses between the specialist and generalist mites.

**FIGURE 4:**
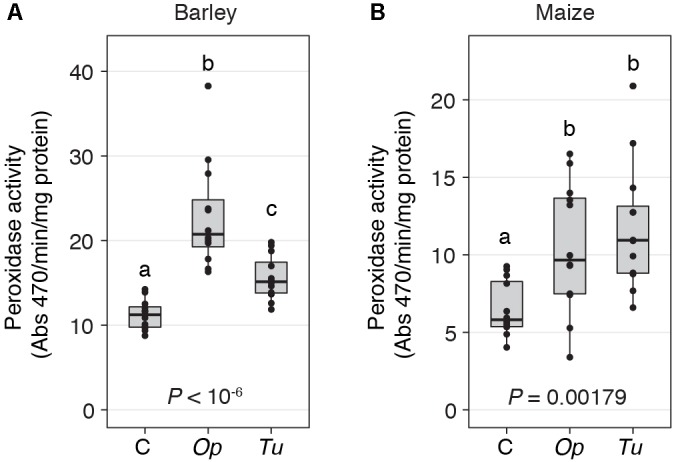
Peroxidase activity increases in response to spider mite herbivory. Peroxidase activity in response to *O. pratensis* (*Op*) and *T. urticae* (*Tu*) herbivory at 24 h in barley **(A)** and maize **(B)**. *P*-values are from an ANOVA, and different letters reflect significant differences among controls (C) and the treatments (*P* < 0.05, Tukey’s HSD test). In the boxplots, circles represent individual data points.

Nonetheless, at the quantitative level, *O. pratensis* induced modestly stronger transcriptomic responses than did *T. urticae*, as assessed by the magnitude of fold changes, in barley at 24 h (**Figure 3B**). Likewise, in maize at the 2-h time point, a similar pattern was observed for the grass specialist (**Figure 3C**). These trends were also apparent in measurements of the activity of peroxidase, which has been reported to increase in response to *T. urticae* herbivory in several dicots (Hildebrand et al., 1986; Liang et al., 2017). In both barley and maize at 24 h, herbivory by both *O. pratensis* and *T. urticae* elevated peroxidase activity relative to control leaves (*P* < 0.05, ANOVA and Tukey’s HSD test; **Figure 4**). Paralleling the difference in the relative magnitude observed for gene expression responses in barley (**Figure 3B**), including for genes encoding peroxidase enzymes (**Supplementary Figure 4**), *O. pratensis* induced significantly greater peroxidase activity compared to *T. urticae* at 24 h (**Figure 4A**).

### Comparison of Plant Responses Induced by Mites to Those Induced by Mechanical Wounding

While mechanical wounding cannot fully replicate the intricacies of physical damage caused by herbivores (Howe and Jander, 2008), our wounding treatment, which consisted of gently pressing leaf blades with sandpaper, mimicked the dispersed nature of spider mite damage to plant leaves (**Figure 1A** and **Supplementary Figure 2**). As assessed by PCAs, at 2 h wounding treatments in both barley and maize clustered separately and further from controls compared to the mite treatments, and more genes were differentially regulated (**Figure 2**). In contrast, at 24 h, biological replicates for wounding treatments clustered nearer control samples in both barley and maize, and fewer genes were detected as differentially expressed compared to wounding at 2 h, or to *O. pratensis* and *T. urticae* feeding at 24 h (**Figure 2**). However, despite differences in the number of DEGs between the wounding and mite feeding treatments, the direction of changes in gene expression and their fold-induction were correlated in both barley and maize between wounding and mite feeding at 2 as well as 24 h (*R*^2^-values between 0.12 and 0.52, with all *P*-values < 10^-15^; **Supplementary Figure 5**).

### Defense Pathways That Respond to Mite Herbivory in Barley and Maize

**FIGURE 5:**
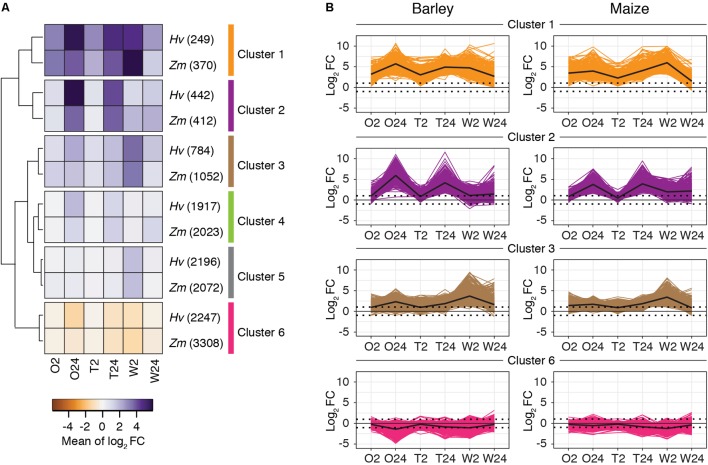
Global patterns of gene expression in response to spider mite herbivory and wounding are similar between barley and maize. **(A)** By plant host, *k*-means clusters (*k* = 6) for DEGs (FDR-adjusted *P*-value of 0.01, absolute value log_2_ fold change cutoff of 1) between treatments and controls (gene numbers in clusters are given in parentheses). For inclusion in the analysis for either barley or maize, a gene had to be significantly differentially expressed in at least one treatment relative to the respective control. Shown is a hierarchical clustering of the barley and maize clusters based on the mean expression values of each cluster. **(B)** Gene expression profiles for barley and maize genes in clusters 1, 2, 3, and 6 as indicated (for clusters 4 and 5, see **Supplementary Figure 6**). Lines reflect expression changes (log_2_ fold change relative to controls) for individual genes across treatments. The mean changes of expression by cluster are shown with black lines. *Hv*, *Hordeum vulgare* (barley); *Zm*, *Zea mays* (maize); O2 and O24, *O. pratensis* herbivory at 2 and 24 h; T2 and T24, *T. urticae* herbivory at 2 and 24 h; and W2 and W24, wounding at 2 and 24 h.

To further characterize gene expression changes across treatments, time points, and plant hosts, we performed *k-*means clustering in barley and maize using expression levels for all genes significantly differentially expressed in at least one treatment. For *k* = 6, and as assessed subsequently with hierarchical clustering (**Figure 5A**), analogous clusters with similar patterns of RNA abundances were readily observed between barley and maize (**Figure 5B**). Clusters 1–5 contain genes that were mostly upregulated in response to either herbivory or wounding, whereas genes in cluster 6 were mostly downregulated (**Figures 5A,B**; clusters 4 and 5, which contain genes with relatively modest fold changes, are shown in **Supplementary Figure 6**).

To relate genes in clusters to biological functions, we performed a GO enrichment analysis for BPs as implemented in the topGO package (Alexa et al., 2006). Significantly enriched GO terms for all clusters for both barley and maize are given in **Supplementary Tables 1**, **2**, respectively. This analysis revealed that herbivory by *O. pratensis* and *T. urticae*, as well as wounding, led to a general switch in transcriptomic programs from processes such as development to those associated with response to the environment. For example, cluster 6, representing mostly downregulated genes in the treatments, was enriched for GO terms related to photosynthesis and development. In contrast, clusters with genes upregulated in response to mite herbivory or wounding, such as clusters 1 and 2, were enriched for GO terms associated with abiotic or biotic defense responses in barley, maize, or both. These included ontology terms such as “JA biosynthetic process,” “response to JA,” “response to SA,” “response to fungus,” “response to wounding,” and “hyperosmotic salinity response.” Of the genes in the six clusters, fold changes for those in cluster 1 were most dramatic with respect to mite feeding across the time course (strong upregulation at 2 h, and even greater upregulation at 24 h). Genes in this cluster were also highly upregulated in response to wounding, especially at 2 h. A reduced correspondence between the magnitude of gene expression changes to mite feeding and wounding was observed in other clusters (e.g., clusters 2 and 3).

We also examined whether the composition of analogous clusters between barley and maize was enriched in orthologous genes, which would suggest common response pathways. To do this, we identified 14,087 genes as orthologs between barley and maize as assessed with a reciprocal best BLASTP hit analysis. For each of clusters 1–6, orthologous gene pairs were enriched between barley and maize (all *P-*values < 10^-12^ as determined with hypergeometric tests; **Supplementary Table 3**).

### Genes for JA Biosynthesis and Signaling Respond Rapidly to Mite Herbivores and Wounding

**FIGURE 6:**
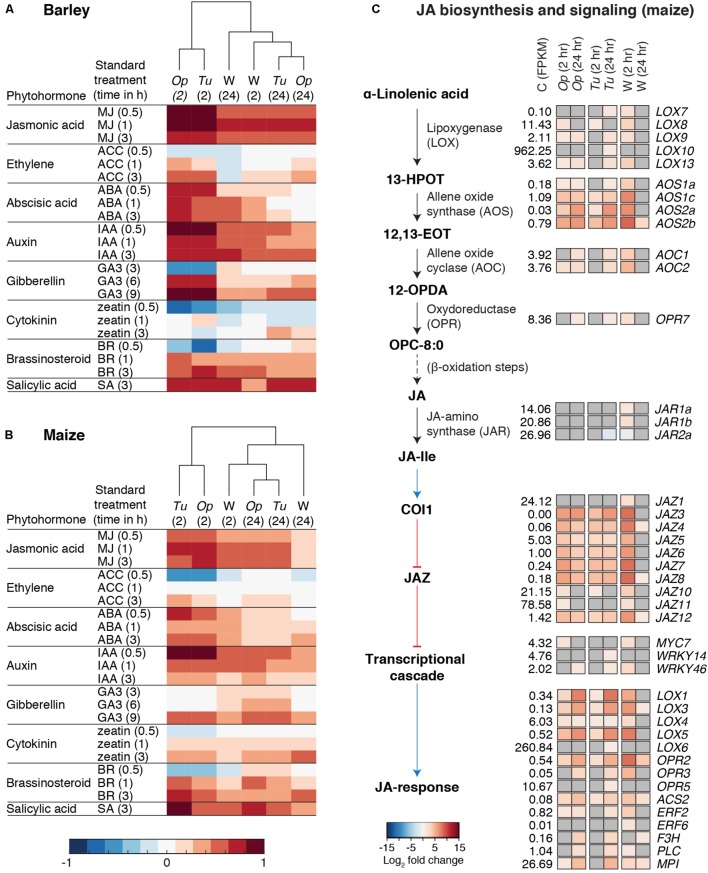
Phytohormone responses to spider mite herbivory and wounding. **(A,B)** Hormonometer analyses for plant hormone signatures based on transcriptomic responses of barley **(A)** and maize **(B)** to *O. pratensis* (*Op*) and *T. urticae* (*Tu*) herbivory as well as wounding (W) at 2 and 24 hours (hr) as indicated in parentheses. The colors indicate similarity between the herbivory/wounding treatments and a particular hormone response (blue and red for negative and positive correlations, respectively, see bottom). MJ: methyl jasmonate; ACC: 1-aminocyclopropane-1-carboxylic acid (a precursor of ethylene); ABA: abscisic acid; IAA: indole-3-acetic acid; GA3: gibberellin A3; BR: brassinosteroid; and SA: salicylic acid. **(C)** Schematic of the JA biosynthesis and signaling pathway after Borrego and Kolomiets (2016) (left) with heat maps of log_2_ fold changes for DEGs, right (FDR adjusted *P*-value of 0.01, absolute value log_2_ fold change cutoff of 1). Upregulation or downregulation of marker genes for JA signaling is given in red and blue, respectively, see bottom, for treatments as compared to the control (only genes differentially regulated in at least one contrast are shown). The mean expression level of each gene among control replicates, as assessed by FPKM values, is as indicated (C, control). Where no differential expression was observed for a given gene and treatment, cells are shaded gray. Black arrows indicate chemical transformations (solid: one reaction; dashed: multiple reactions) and blue and red arrows indicate signal transduction (activation and inhibition, respectively).

The GO enrichment analysis suggested a prominent role for phytohormone signaling in responses to both mite herbivores and wounding. Therefore, we adapted the Hormonometer tool (Volodarsky et al., 2009) to relate mite- and wounding-induced changes in gene expression to those induced by diverse phytohormones. As displayed in dendrograms for both plant hosts (**Figures 6A,B**), changes in most phytohormone response pathways were readily apparent by 2 h in response to herbivory by *O. pratensis* and *T. urticae*, as well as wounding. In general, patterns of up- or downregulation for plant hormone responsive genes were similar between barley and maize, and at 24 h responses were generally attenuated. Response genes for JA, SA, ABA, and auxin were most dramatically upregulated, while response genes associated with ethylene, GA, cytokinin, and brassinosteroid signaling were either downregulated, variable, or largely unaffected.

Because of the strong JA responses, and functional-genetic studies demonstrating a role for JA signaling in deterring *T. urticae* feeding on *A. thaliana* and tomato (Zhurov et al., 2014; Martel et al., 2015), we characterized transcriptomic responses of this pathway further. While the JA pathway is well characterized in dicots, less about it is known in monocots. However, candidate genes for JA biosynthesis, signal transduction, and mediation of transcriptional changes have been identified in maize (Borrego and Kolomiets, 2016), and genetic studies provide functional evidence for the involvement of several of these genes in JA biosynthesis and signaling (Yan et al., 2012; Tzin et al., 2017). Initially, we focused our analysis on the JA pathway in maize (**Figure 6C**), describing general patterns of responses to both mite species as induced gene expression is highly correlated between them (**Figure 3**).

Lipoxygenases (LOXs) catalyze the production of oxylipins, which are involved in multiple aspects of plant defense (Borrego and Kolomiets, 2016). The first step in the JA biosynthesis pathway is the production of the oxylipin 13(*S*)-hydroperoxylinolenate (13-HPOT) from α-linolenic acid. Putative LOXs for catalyzing this reaction belong to the 13-LOX group, of which maize has five (*LOX7*, *8*, *9*, *10*, *11*, *13*) (Borrego and Kolomiets, 2016). Of these, all but *LOX11* were induced at one or more time points, albeit with modest fold changes. As assessed by normalized expression values (FPKMs), basal levels of *LOX10* were ∼90-fold higher than for other 13-LOX genes, consistent with its role in the production of green leaf volatiles (Christensen et al., 2013), which are abundant in grasses. The next two steps in JA synthesis involve the conversion of 13-HPOT to 12,13(*S*)-epoxylinolenic acid (12,13-EOT), followed by cyclization to yield 12-oxo-phytodienoic acid (12-OPDA) (Borrego and Kolomiets, 2016). Differential expression was also observed for putative genes mediating these steps, including the upregulation of four *ALLENE OXIDE SYNTHASE* (*AOS*) genes and two *ALLENE OXIDE CYCLASE* (*AOC*) genes, respectively. As compared to *13-LOX* and *AOC* genes, the *AOS* genes were more highly upregulated at both 2- and 24-h time points. This included genes in the *AOS1* clade, but also genes in clade 2 for which a role in JA biosynthesis is uncertain (Borrego and Kolomiets, 2016). The next step in JA synthesis involves the conversion of 12-OPDA to 3-oxo-2-(*cis*-2′-pentenyl)-cyclopentane-1-octanoic acid (OPC-8:0), which is mediated by oxophytodienoate reductase (OPR). In maize, this step requires the products of *OPR7* and *OPR8*, for which JA levels are dramatically reduced in tissues of the double mutant, including in leaves (Yan et al., 2012). One of these genes, *OPR7*, was modestly upregulated at 24 h in response to mite herbivory. The conjugation of JA to isoleucine, which is carried out by the JASMONATE RESISTANT1 (JAR1) protein in *A. thaliana* (Staswick, 2002), produces JA-Ile, the most biologically active form of JA in plants (Koo and Howe, 2012). One maize gene encoding a JAR protein, *JAR2a*, was weakly downregulated at 24 h after *T. urticae* feeding, mirroring its downregulation in response to *S. exigua* herbivory (Tzin et al., 2017).

To assess JA signaling, we examined expression changes for genes downstream of JA-Ile, including ones that were identified as not being inducible (or being less induced) in response to wounding in *OPR7*/*8* double mutant maize plants (Yan et al., 2012; Borrego and Kolomiets, 2016). Many of these genes increased in expression in response to mite feeding, albeit moderately (i.e., the transcriptional regulators *MYC7*, *WRKY14*, and *WRKY46*). Nevertheless, genes in several families showed dramatic upregulation. For instance, *JAZ3*, *4*, *5*, *6*, *7*, *8*, and *12* were strongly upregulated at both the 2- and 24-h time points. Further, members of the 9-LOX clade, including *LOX3*, *4*, and *5*, were also upregulated at both time points, with *LOX3* and *5* exhibiting strong upregulation at 24 h. *LOX1*, which encodes a dual activity lipoxygenase (13-LOX and 9-LOX) of unknown function (Borrego and Kolomiets, 2016), was also upregulated at both time points.

Collectively, gene expression changes for JA biosynthesis and downstream responses were induced similarly between *O. pratensis* and *T. urticae*, with patterns at 2 and 24 h essentially identical where strong differential gene expression was observed (e.g., for most *LOX*, *AOS*, and *JAZ* genes). Further, responses to wounding at 2 h closely mirrored those of responses to mite herbivory (**Figure 6C**). While we focused on the JA pathway in maize, for which more is known, an analysis of putative orthologs in barley revealed similar responses (**Supplementary Figure 7**). Finally, mirroring our analysis of genes involved in JA signaling, we performed a similar one for SA, another key plant hormone mediating biotic interactions. Many candidate genes for SA biosynthesis (Martel et al., 2015; Tzin et al., 2015a) were upregulated in response to mite herbivory and wounding at both the 2- and 24-h time points. Examples from maize included genes encoding putative prephenate dehydratases (*GRMZM2G437912* and *GRMZM2G125923)*, Phe ammonia-lyase *(GRMZM2G063917)*, and *trans*-cinnamate 4-monooxygenases (*GRMZM2G147245* and *GRMZM2G010468)* (**Supplementary Figure 8**).

### Upregulation of Defensive Proteins

Expression of protease inhibitors and chitinases has been shown to retard the growth of insects, spider mites, or both (Lawrence and Novak, 2006; Carrillo et al., 2011; Santamaría et al., 2012), and about half of cysteine protease inhibitors (cystatins) and serine protease inhibitors were expressed in barley or maize leaves in controls or treatments (**Supplementary Data Sheets 3**, **4**, respectively). In barley, no cystatin genes changed in expression in response to herbivory by *O. pratensis* or *T. urticae*, although two genes were moderately upregulated in response to wounding at 2 h (**Supplementary Figure 9A**). In contrast, eight maize cystatin genes were modestly upregulated, primarily at 24 h, in response to *O. pratensis* or *T. urticae* herbivory (**Supplementary Figure 9A**). Most of these were also upregulated at either 2 or 24 h in response to wounding. As opposed to the pattern observed for cystatins, higher-fold upregulation of serine protease inhibitors was observed in leaves of both plant hosts in response to feeding by *O. pratensis* and *T. urticae*. Among the upregulated genes were several previously reported to be responsive to wounding or JA signaling, including *WOUND INDUCED PROTEIN1* (*WPI*) and *MAIZE PROTEASE INHIBITOR* (*MPI*) (Eckelkamp et al., 1993; Rohrmeier and Lehle, 1993; Tamayo et al., 2000) (**Supplementary Figure 9B**). A pattern shared with the cystatins in maize was that serine protease inhibitor genes were upregulated at the 24-h time point (or if they were significantly induced at 2 h, induction was higher at 24 h). Most serine protease inhibitors induced by mite herbivory were upregulated by wounding at 2 or 24 h. Twenty-one chitinase genes changed in expression in response to herbivory by *O. pratensis* or *T. urticae*, or wounding, in maize (**Supplementary Figure 10A**). The majority of genes were upregulated, many at 24 h only, although *GRMZM2G129189*, *GRMZM2G145461*, and *GRMZM2G162359* were induced at both time points. The upregulated genes belonged to several glycoside hydrolase families that hydrolyze chitin (GH-18 and GH-19) (Hawkins et al., 2015). A similar pattern of dynamic responses of chitinase genes to mite herbivory and wounding was also apparent for barley (**Supplementary Figure 10B**).

### A Functional Benzoxazinoid Pathway Deters the Generalist but Not the Specialist Mite Herbivore

**FIGURE 7:**
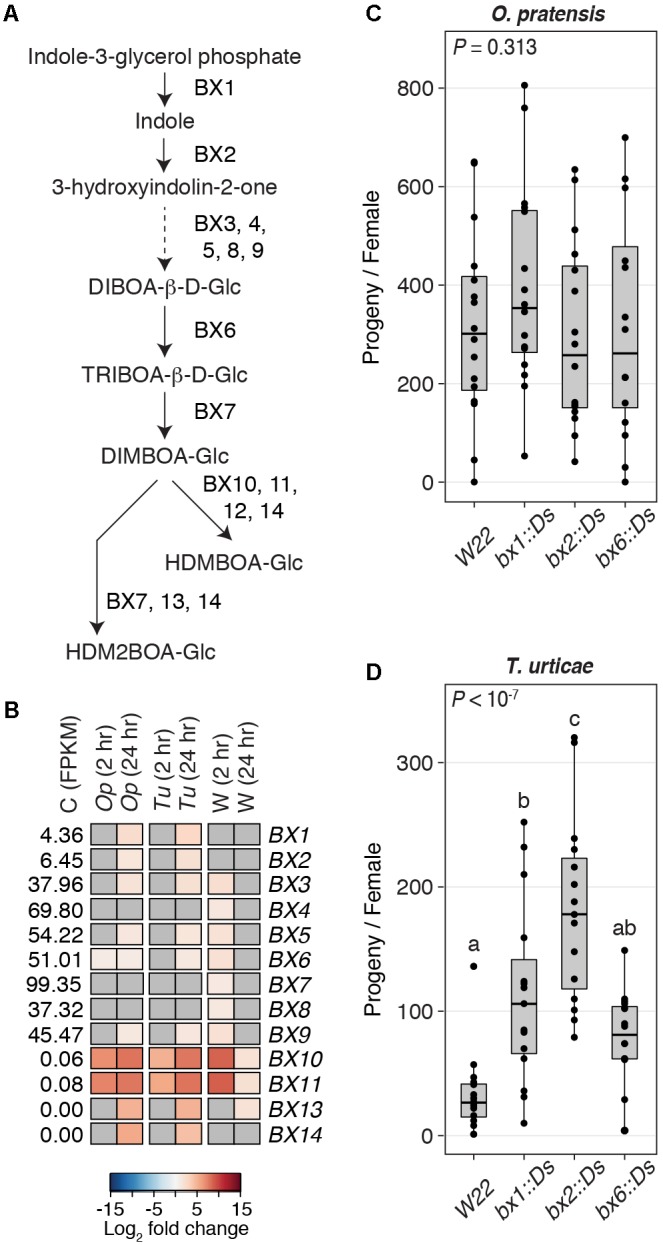
A role for benzoxazinoids in deterring the generalist spider mite, *T. urticae*. **(A)** Biosynthesis pathway for benzoxazinoids after Tzin et al. (2017). Arrows indicate chemical transformations (solid lines, one reaction; dashed line, multiple reactions). **(B)** Heat maps for DEGs (FDR-adjusted *P*-value of 0.01, absolute value log_2_ fold change cutoff of 1) in the biosynthesis pathway for benzoxazinoids in response to herbivory by *O. pratensis* (*Op*) and *T. urticae* (*Tu*) or to wounding (W) at 2 or 24 hours (hr) as indicated. For the control samples (C), the mean expression level among replicates of each gene, as assessed by FPKM values, is given. The colors correspond to log_2_ fold changes of individual genes in each treatment (blue, downregulation; red, upregulation). Where genes were not significantly differentially expressed, cells are gray. **(C,D)** Boxplots showing the number of progeny produced by *O. pratensis* and *T. urticae*, respectively, on W22 (wild-type) and homozygous Ds transposon insertion mutants in *BX1*, *BX2*, and *BX6* on the W22 background. Circles represent individual data points. *P*-values were determined by ANOVA, with letters indicating significant differences among contrasts (*P* < 0.05, Tukey’s HSD test).

Domesticated varieties of barley, including the cultivar Morex used in our study, lack benzoxazinoids (Glawischnig et al., 1999; Niemeyer, 2009). In maize, however, at least 14 genes are involved in the benzoxazinoid biosynthesis pathway, and several participate at multiple steps, as shown in **Figure 7A** (the schematic of the pathway is after Tzin et al., 2017). In B73, the *BX12* gene is disrupted, although HDMBOA-Glc can still be produced (Meihls et al., 2013).

In all cases, genes for the synthesis of DIMBOA-Glc were expressed in control B73 tissue, with *BX1* and *BX2* having the lowest expression levels (**Figure 7B**). In response to herbivory by either *O. pratensis* or *T. urticae*, six of the genes needed for synthesis of DIMBOA-Glc were induced weakly at 24 h (**Figure 7B**; *BX6* was also differentially expressed at 2 h in response to *O. pratensis*, albeit with a small fold change). Seven of these genes were also induced weakly by wounding at 2 h, but none remained upregulated at 24 h post-wounding. In contrast to genes needed for DIMBOA-Glc synthesis, those for the modification of DIMBOA-Glc to produce HDMBOA-Glc and HDM2BOA-Glc had very low basal expression levels in control tissue (**Figure 7B**). However, two genes involved in the production of HDMBOA-Glc, *BX10* and *BX11*, were dramatically upregulated in response to *O. pratensis* and *T. urticae* herbivory as well as wounding at 2 h, and remained strongly induced at 24 h. Two other genes, *BX13* and *BX14* that are needed for the production of HDM2BOA-Glc, were also induced by mite herbivory, but with upregulation only observed at 24 h; in response to wounding, *BX13* was also upregulated at 24 h, albeit only moderately. We also compared the relative upregulation of benzoxazinoid biosynthesis genes in response to spider mite herbivory at 24 h to those reported for *S. exigua* feeding at the same time point (Tzin et al., 2017). Although different stage plants were used (see the section “Discussion”), genes for the synthesis of DIMBOA-Glc (e.g., *BX1* and *BX2*) were less strongly induced by spider mites, while those for the conversion of DIMBOA-Glu to HDMBOA-Glc and HDM2BOA-Glc (especially *BX10* and *BX11*) were induced strongly by both mites as well as the caterpillar herbivore (**Supplementary Table 4**).

In the W22 maize inbred, *Ds* transposon insertions have been recovered in three genes responsible for DIMBOA-Glc synthesis – *BX1*, *BX2*, and *BX6* (Tzin et al., 2015a). For *O. pratensis*, reproductive performance, as assessed by the number of progeny per female, did not differ significantly following infestation of wild-type and *bx* mutant plants (ANOVA, *P* = 0.313; **Figure 7C**). In contrast, for *T. urticae* stark differences were observed (ANOVA, *P* < 10^-7^; **Figure 7D**). While the number of *T. urticae* progeny did not differ between wild-type (W22) and *bx6::Ds* plants, significantly more progeny were observed on both *bx1::Ds* and *bx2::Ds* plants compared to wild-type, and significantly more progeny were observed on *bx2::Ds* compared to *bx1::Ds* plants (*P* < 0.05 after correction for multiple comparisons with Tukey’s HSD method).

### Genes Required for the Production of Volatile Plant Compounds Are Induced by Mite Herbivory

In addition to green leaf volatiles and methyl salicylate (MeSA) – volatile organic compounds whose biosynthesis genes were either constitutively expressed or induced by mite herbivory (**Figure 6** and **Supplementary Figure 8**) – terpenes are well-characterized herbivore-induced plant volatiles (HIPVs) that mediate plant responses to herbivory, including indirect defenses (Kessler and Baldwin, 2002; Singh and Sharma, 2015). In maize, the *TERPENE SYNTHASE* (*TPS*) genes *TPS10*, *TPS2*, and *TPS3* were highly induced at 2 h in response to *O. pratensis* and *T. urticae*, as well as by wounding (each gene remained induced at 24 h; **Supplementary Figure 11**). Additionally, 14 other putative maize *TPS* genes were induced with lesser fold changes, primarily at 24 h, in response to mite herbivory, or at 2 h in response to wounding. Patterns were similar for barley *TPS* genes including *MLOC_56812* and *MLOC_76989*, which were strongly induced by *O. pratensis* and *T. urticae* herbivory at both time points, and *MLOC_13618* at 24 h. In maize, TPS2 and TPS10 synthesize multiple products. For TPS2, two products are (E)-nerolidol and (E,E)-geranyllinalool, which are subsequently converted to the homoterpenes (E)-3,8-dimethyl-1,4,7-nonatriene (DMNT) and (E,E)-4,8,12-trimethyltrideca-1,3,7,11-tetraene (TMTT), respectively. Recently, Richter et al. (2016) implicated the cytochrome P450 genes *CYP92C5* and *CYP92C6* in the final step for the production of DMNT and TMTT. Both genes were induced in response to mite herbivory at 2 or 24 h, and *CYP92C5* was upregulated by wounding at 2 h (**Supplementary Figure 11**).

In maize, *IGL* is responsible for volatile indole, which is inducible by an insect trigger, and was recently shown to prime defenses within and between maize plants (Erb et al., 2015). In response to herbivory by *O. pratensis* and *T. urticae* in maize, *IGL* was modestly upregulated at 24 h (log_2_ fold changes ∼2) but not at 2 h; in the wounding treatment, *IGL* was upregulated by a similar fold change at 2 h, but was not significantly changed in expression at 24 h (**Supplementary Data Sheet 4**).

## Discussion

Whether specialist and generalist herbivores induce different plant responses, and if so, to what degree the herbivore or the plant benefits, has attracted long-standing interest (Ali and Agrawal, 2012). Experimental approaches to tackle these questions have often been confounded by factors including differences in feeding guild, comparisons between phylogenetically divergent herbivores, inclusion of single plant hosts, variation in the extent of tissue damage, and the lack of an expectation of plant defense responses in the absence of (potential) manipulation by herbivores (Ali and Agrawal, 2012). Our study addresses several of these confounding factors. While *O. pratensis* and *T. urticae* are in different genera, they are closely related within the family Tetranychidae (Matsuda et al., 2014), differ little in size and morphology, and cause similar levels of tissue damage on barley and maize leaves. At the cellular level, we found that both species feed on mesophyll cells. This pattern of damage agrees with reports for *Tetranychus* mites in dicots, where empty mesophyll cells were reported at feeding sites (Bensoussan et al., 2016, and references therein). Further, Bensoussan et al. (2016) showed that adult *T. urticae* females feed on mesophyll cells in *A. thaliana* and bean by inserting their stylets either through stomata or between epidermal pavement cells. Our observation of a seemingly intact epidermis overlying empty mesophyll cells in barley suggests that spider mites use a similar feeding mechanism in grasses.

Although spider mites cause less dramatic tissue damage than insects like caterpillars, both *O. pratensis* and *T. urticae* induced pronounced changes in gene expression and peroxidase activity in barley and maize. Strikingly, the DEG sets induced by the two mite species in both plant hosts were similar in composition as well as in the direction (up- or downregulation) and magnitude of fold changes. For the latter, several modest differences in magnitude were apparent. For instance, *O. pratensis* induced a slightly stronger transcriptomic response than *T. urticae* at 24 h in barley, and at 2 h in maize. Whether these differences reflect modulation of plant responses by either herbivore, or alternatively arise from behavioral differences or other factors, is not clear. Nevertheless, neither mite species appears to differentially manipulate barley or maize defenses to a great extent (or alternatively, the two plants do not distinguish between the two mite species to mount different responses). A caveat is that our study does not rule out the possibility that manipulation could occur post-transcriptionally. It should further be noted that our study used only one strain of *T. urticae*, which we maintained on bean until several generations before collecting barley and maize transcriptomic data. Several experimental studies have documented that the generalist *T. urticae* can adapt to its hosts when continuously maintained over many generations (Gould, 1979; Fry, 1989; Agrawal, 2000; Magalhães et al., 2007). If *T. urticae* (and possibly *O. pratensis*) populations adapt to specific grass hosts, potentially by gaining the ability to modulate plant defenses, is not known. Therefore, whether our findings generalize to all *T. urticae* and *O. pratensis* populations is an outstanding question. However, our experimental design mimicked the agriculturally relevant setting for cereal crops in which spider mites invade fields from weeds or other crops, persist during the growing season for a modest number of generations, and then move to other hosts for overwintering (Margolies and Kennedy, 1985).

Our findings of similar barley and maize responses to *O. pratensis* and *T. urticae* herbivory do not rule out the possibility that both suppress plant responses similarly. To test this, as well as to understand how grasses perceive mite herbivores, we also included a wounding treatment against which potential suppression of defenses could be assessed (Howe and Jander, 2008; Ali and Agrawal, 2012). The utility of a wounding treatment depends largely on how well the treatment mimics patterns of herbivore tissue damage (Mithofer et al., 2005). In the case of mites, mimicking mechanical damage to individual mesophyll cells is not possible. Further, our wounding treatments did not replicate the continuous nature of mite feeding, and consisted instead of marked and instantaneous tissue damage at the beginning of treatments. Despite these caveats, in both barley and maize, genes that responded most strongly to herbivory at both 2 and 24 h also responded strongly to wounding alone. This suggests that grasses readily perceive physical tissue damage by spider mites and mount strong defense responses. Additionally, for many known defensive genes, it was striking that expression changes induced by wounding (up- or downregulation, as well as magnitude of fold changes) were similar to those induced by mite feeding. Therefore, within the limits of our experimental design, we found no obvious signs that *O. pratensis* or *T. urticae* suppress plant defenses associated with tissue disruption.

In both barley and maize, reprogramming of the transcriptome in response to mite feeding was dynamic over a 24-h time period. At 2 h, most DEGs were upregulated, including genes associated with JA and other phytohormone signaling, the production of some specialized metabolites, and the synthesis of HIPVs. More dramatic changes in gene expression were observed at 24 h, including a mix of up- and downregulated genes. These dynamics resemble those reported previously in studies with *T. urticae* in dicots including *A. thaliana*, tomato, and grapevine (Zhurov et al., 2014; Martel et al., 2015; Díaz-Riquelme et al., 2016). Recently, Santamaría et al. (2018) examined transcriptomic responses to *T. urticae* in barley in a design that examined both biotic and abiotic stresses. Although their experimental design differed markedly from ours, with transcriptomic responses assessed at long time points (RNA-seq data were collected after 7 days of herbivore exposure), they also observed upregulation of genes associated with JA biosynthesis and signaling. In maize, our findings are also consistent with those of Szczepaniec et al. (2013), who found by reverse transcription quantitative polymerase chain reaction that several marker genes for JA and SA signaling were upregulated after 3 days of herbivory by *T. urticae*. More generally, at the whole transcriptome level for maize, the transcriptomic changes we observed following herbivory by spider mites are also similar to those reported for herbivory by the caterpillar *S. exigua* (Tzin et al., 2017). These similarities encompassed rapid induction of diverse LOX genes, including but not limited to those involved in JA biosynthesis and signaling. This suggests that chewing insects and mesophyll-feeding mites induce globally similar transcriptomic responses even though patterns of tissue damage differ radically. The downregulation of genes involved in photosynthesis that we observed in response to mite herbivory has been reported to be a general response to biotic stress (Bilgin et al., 2010).

We found that genes in several families encoding defensive proteins, including protease inhibitors and chitinases, were upregulated in our study. Transgenic expression of plant protease inhibitors has been shown to reduce *T. urticae*’s performance on several plant hosts (Carrillo et al., 2011; Santamaría et al., 2012). The same was also observed for transgenic expression of a chitinase, albeit from an insect source (McCafferty et al., 2006), although it should be noted that plant-produced chitinases in the frass of *S. frugiperda* were found to suppress plant defenses and favor the herbivore (Ray et al., 2016). These transgenic studies relied on overexpression, however, and the extent to which endogenous production of protease inhibitors or chitinases in barley and maize leaves impacts spider mites is not known. In insects, protease overexpression or expression of alternative proteases is one route to overcome ingested, plant-produced inhibitors (e.g., Kuwar et al., 2015). This mechanism is likely relevant for *T. urticae*, as sequencing of the *T. urticae* genome revealed expansions of protease families, some of which were found to be highly induced upon plant host shifts (Grbić et al., 2011). Whether protease families are expanded in *O. pratensis*, or whether this specialist has evolved specialized digestive proteases to overcome the inhibitors produced by its hosts in Poaceae, is an outstanding question.

Beyond defensive proteins, HIPVs released from feeding sites on grass leaves may play important roles in indirect defenses against mites. In both barley and maize, genes for the synthesis of SA (the precursor to the volatile MeSA), as well as for the synthesis of terpenes, were upregulated in response to mite herbivory. These can serve as cues to predators of spider mites, which include predatory mites as well as winged ladybird beetles (family Coccinellidae). Within Coccinellidae, minute species of the tribe Stethorini feed primarily on mites in the Tetranychidae family (Biddinger et al., 2009). For instance, *Parastethorus nigripes* is an introduced species that has established on *O. pratensis* on maize in the southern United States (Pollock and Michels, 2002). In Y-tube olfactometer experiments, predatory mites were attracted by MeSA or terpenes (De Boer et al., 2004; Kappers et al., 2005), and synthetic MeSA was shown to attract a Stethorini species (James and Price, 2004). *IGL* was also modestly upregulated in maize in response to herbivory, suggesting that volatile indole is released at mite feeding sites. While indole is a priming agent in maize (Erb et al., 2015), whether it also serves as an attractant for predators of spider mites is unknown.

Apart from defensive proteins and HIPVs, several specialized compounds are likely to play roles in defense against mite herbivores. Mirroring findings reported for herbivory by *S. exigua* (Tzin et al., 2017), genes encoding 9-LOX proteins were rapidly induced by mite feeding. Unlike 13-LOX proteins involved in JA synthesis or the production of green leaf volatiles, LOX3, 4, and 5 belong to the 9-LOX clade, which likely has diverse functions including the production of “death acid” compounds; 10-OPEA, one such compound, was shown to reduce the performance of fungal pathogens and the lepidopteran herbivore *Helicoverpa zea* (Christensen et al., 2015). More recently, it was shown that *S. exigua* growth increased on maize plants with transposon insertions in *LOX4* as compared to wild-type plants (Woldemariam et al., 2018). These findings, coupled with the upregulation of *LOX3*, *4*, and *5* that we observed in our study, suggest that members of the 9-LOX clade should also be assessed for roles in deterring spider mites.

Additionally, we observed modest upregulation of genes involved in the synthesis of DIMBOA-Glc. However, we found dramatic and rapid upregulation of *BX10* and *BX11* that modify DIMBOA-Glc to produce HDMBOA-Glc, which has been associated with resistance to multiple lepidopteran species that feed on maize (Glauser et al., 2011; Tzin et al., 2015b). In contrast, we did not observe as rapid or as strong an induction for genes needed for the synthesis of HDM2BOA-Glc. This may suggest different transcriptional regulation of the biosynthesis genes for major classes of benzoxazinoids derived from DIMBOA. Further, despite globally similar transcriptomic responses to mite herbivory in our study compared to those reported for *S. exigua* (Tzin et al., 2017), some *BX* genes, especially those required for DIMBOA-Glc and HDM2BOA-Glc synthesis (e.g., *BX1* and *BX14*, respectively), were more strongly induced by *S. exigua*. In Tzin et al.’s (2017) study, younger plants were used for the transcriptomic analysis, and the inducibility of benzoxazinoids has been shown to decrease as maize plants age (Köhler et al., 2015). Whether differences in plant stage, the scope of tissue damage, or other factors explain differences in relative induction for some benzoxazinoid synthesis genes between our study and that of Tzin et al. (2017) warrants additional investigation.

Mutations in the benzoxazinoid pathway in maize allowed us to test if benzoxazinoids deter *O. pratensis* or *T. urticae*, both, or neither. For *T. urticae*, performance was markedly reduced on wild-type plants compared to homozygous *bx1::Ds* and *bx2::Ds* plants. Our finding that *T. urticae* performed better on *bx2::Ds* plants than on *bx1::Ds* plants was unexpected, as both are reported to reduce DIMBOA to the same low level in W22 (Tzin et al., 2017). One possibility is that indole produced by BX1, which would be anticipated to accumulate in *bx2::Ds* mutant plants, negatively affects *T. urticae*. Previously, Tzin et al. (2015a) found that DIMBOA-Glc levels were only modestly reduced (∼70%) in *bx6::Ds* plants, suggesting substantial functional redundancy at the respective step in the benzoxazinoid pathway. This likely explains our finding that mite performance was not significantly different on *bx6::Ds* compared to wild-type plants.

In line with our finding for *T. urticae* and benzoxazinoids in maize, *A. thaliana* plants unable to make indole glucosinolates – a class of specialized compounds in the Brassicaceae – are less resistant to *T. urticae* (Zhurov et al., 2014). Combined with results from our study, this observation is at odds with the supposition, for which there is mixed experimental support in insects, that generalists should be good at suppressing phylogenetically conserved plant defense pathways (like canonical phytohormone signaling upstream of plant family-specific defensive compounds) (Ali and Agrawal, 2012). It is consistent, however, with an important role for detoxification in underlying *T. urticae*’s extreme host range, as supported by the finding of expansions of diverse detoxification genes in this species’ genome (Grbić et al., 2011), and its known ability to detoxify compounds from diverse chemical classes (Van Leeuwen and Dermauw, 2016). However, specialized compounds that typify plant families, like benzoxazinoids in Poaceae, nonetheless appear to be costly for *T. urticae*. It should also be noted that our performance assessments for *T. urticae* on *bx* mutants were performed on young plants, in which levels of benzoxazinoids are expected to be higher than in older plants (Cambier et al., 2000; Köhler et al., 2015). Although benzoxazinoid synthesis can be induced in maize (Köhler et al., 2015; Maag et al., 2016), screens of maize lines for resistance to *T. urticae* have shown that while many lines are resistant when young, resistance is ameliorated or essentially lost in older plants for some, although not all, inbred lines (Tadmor et al., 1999). Therefore, determining the extent to which benzoxazinoids deter herbivory by *T. urticae* in field settings – where infestation and significant plant damage typically occur later in the growing season – requires further study.

In contrast, our characterization of *O. pratensis* performance on wild-type and *bx* mutant plants suggests that *O. pratensis* is not affected by benzoxazinoids. The simplest interpretation of this finding is that the route to specialization for *O. pratensis*, at least with respect to this class of toxic compounds, lies in a specialized mechanism of detoxification or inactivation. Precedence for this comes from insects, where several caterpillars have been shown to enzymatically render ingested benzoxazinoids non-toxic (or less toxic) by glucosylation (Glauser et al., 2011; Maag et al., 2014). Our findings with *O. pratensis* contrast with those of other specialist mite herbivores like *T. evansi* and *A. lycopersici*, for which suppression of host plant defenses has been documented (Glas et al., 2014; Alba et al., 2015). Thus, multiple paths to specialization appear to have been taken by different herbivorous mites.

## Concluding Remarks and Future Directions

Our findings revealed dynamic yet highly correlated transcriptomic responses of two major cereal crops to two spider mite herbivores. Further, the plant responses resembled those observed for wounding, a physical component of herbivory. Taken together, these results suggest that neither mite species manipulates defense responses of these grasses, nor that the grass hosts distinguish between the generalist and specialist mites to initiate different (and potentially adaptive) defensive programs. Nevertheless, we found that the generalist *T. urticae* is negatively impacted by the benzoxazinoid defenses of maize. Our study included the widely used maize inbreds B73 and W22, both of which are readily fed upon by *T. urticae*. These lines are representative of most maize inbred lines in that they are comparatively sensitive to *T. urticae*; nevertheless, a small number of lines have been reported to be resistant throughout their ontogeny (Tadmor et al., 1999). Our findings suggest that variation in benzoxazinoid levels or types should be investigated as a potential factor in explaining resistance in maize to *T. urticae*, and intraspecific variation in benzoxazinoid biosynthesis or accumulation in maize has been documented (Meihls et al., 2012, 2013). Further, in agriculture, economic damage to maize from spider mites is typically observed under drought conditions. As we did not include drought stress as a factor in our experimental design, a future challenge will be to test whether this key abiotic stress influences the relative defense responses of cereals to generalist and specialist spider mites. Given the importance of resistant germplasm for breeding programs, these studies should be extended as well to examine maize varieties previously reported to be mite resistant.

## Data Availability

The RNA-seq data sets for barley and maize generated and analyzed for this study are available through Gene Expression Omnibus (GEO) under accession numbers GSE83676 and GSE100121, respectively.

## Author Contributions

HB, RR, and RC conceived the research. HB, RG, RC, SL, AR, GG, and RR performed the experiments. Analysis of the data was performed primarily by HB and RG. HB, RG, and RC assumed primary responsibility for writing the manuscript, with input from all authors.

## Conflict of Interest Statement

The authors declare that the research was conducted in the absence of any commercial or financial relationships that could be construed as a potential conflict of interest.

## Footnotes

1 https://Github.com/chapmanb/bcbio-nextgen
