# Supplementary material for: Generalist and Specialist Mite Herbivores Induce Similar Defense Responses in Maize and Barley but Differ in Susceptibility to Benzoxazinoids
Source: Front Plant Sci. 2018 Aug 21;9:1222. doi: 10.3389/fpls.2018.01222 (PMC6110934; doi:10.3389/fpls.2018.01222)
Supplement: Supplementary file 5 [file Presentation_1.pdf]

Supplementary Material

Generalist and Specialist Mite Herbivores Induce Similar Defense Responses in Maize and Barley but Differ in Susceptibility to Benzoxazinoids

Huyen Bui, Robert Greenhalgh, Alice Ruckert, Gunbharpur S. Gill, Sarah Lee, Ricardo A. Ramirez, Richard M. Clark*

*** Correspondence:** Richard M. Clark: richard.m.clark@utah.edu

1 Supplementary Figures

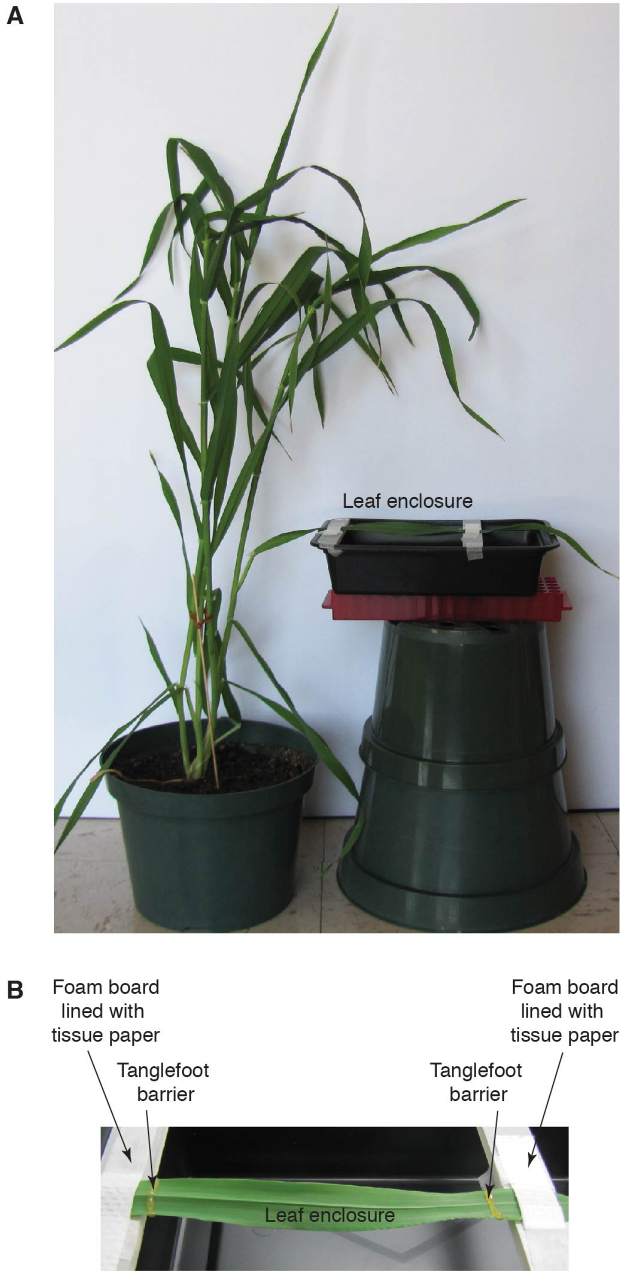

Supplementary Figure 1. Immobilization of barley leaves and leaf enclosures. **(A)** Barley leaves can twist and touch other leaves, making it difficult to establish enclosures and apply mites. Therefore, leaves attached to plants were immobilized as shown. **(B)** Close up of an enclosure. Leaves were immobilized between tissue paper that was held taut by tape flanking enclosures. The actual leaf blades only touched the tissue paper, and gentle pressure was applied to the tissue paper (to prevent wounding). Tanglefoot barriers were internal to the strips of tissue paper over a plastic container and connected on each side of the leaf blades (i.e., mites applied in the middle of enclosures could not disperse to either side of leaves beyond the barriers). A similar design was used for maize, except that no leaf immobilization was required as maize leaves are rigid and well separated.

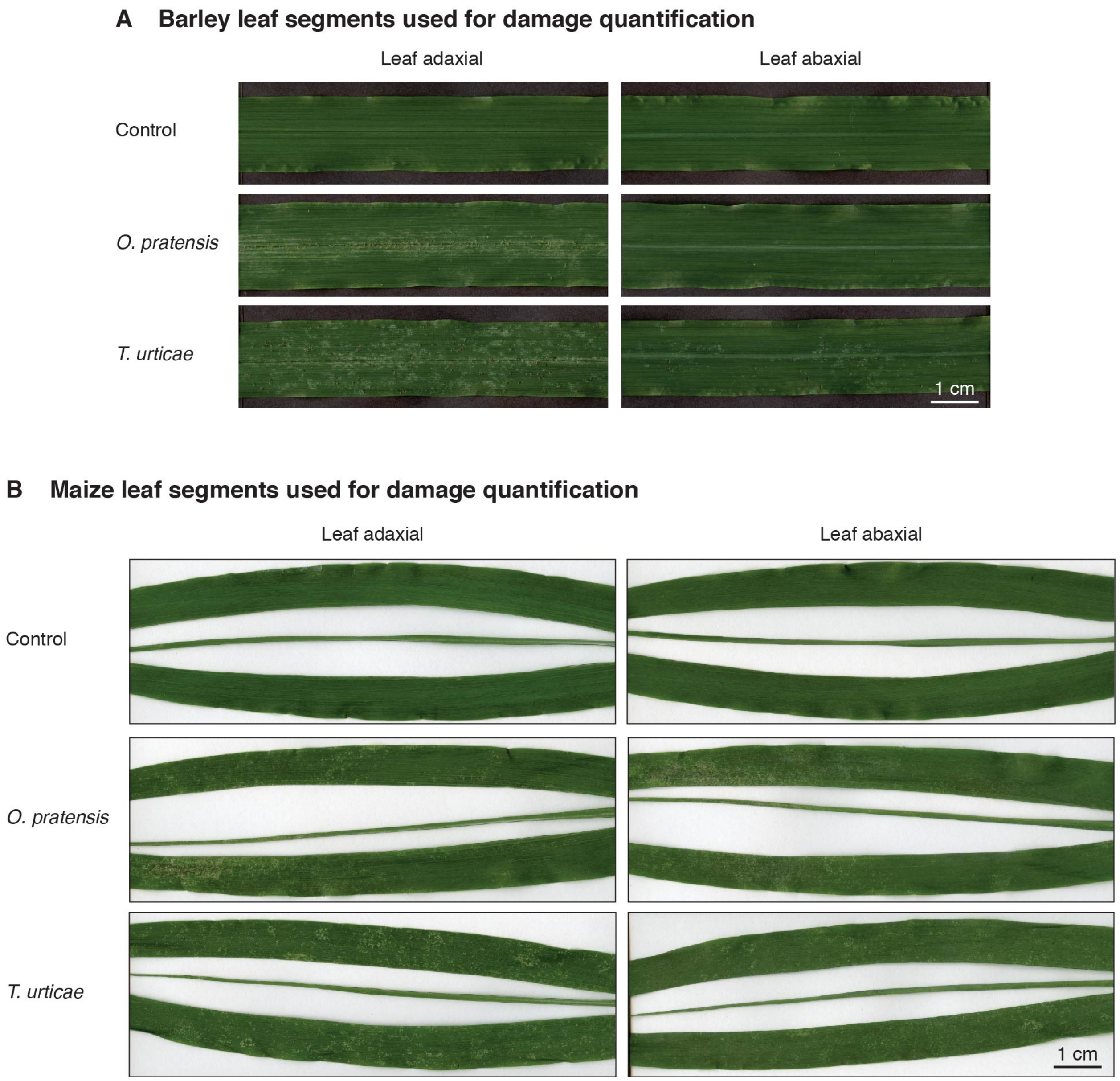

Supplementary Figure 2. Representative scanned images used for quantification of mite feeding damage. **(A)** Representative scans of the adaxial and abaxial blades of barley leaf segments from control leaves and those infested with *O. pratensis* and *T. urticae* for 24 hours as indicated. **(B)** Representative scans of the adaxial and abaxial blades of maize leaf segments from control leaves and those infested with *O. pratensis* and *T. urticae* for 24 hours as indicated. To facilitate scanning, the maize leaf segments were cut along the midrib, and then the midrib and two halves of the leaf blades were pressed flat for scanning. The scale bar is 1 cm.

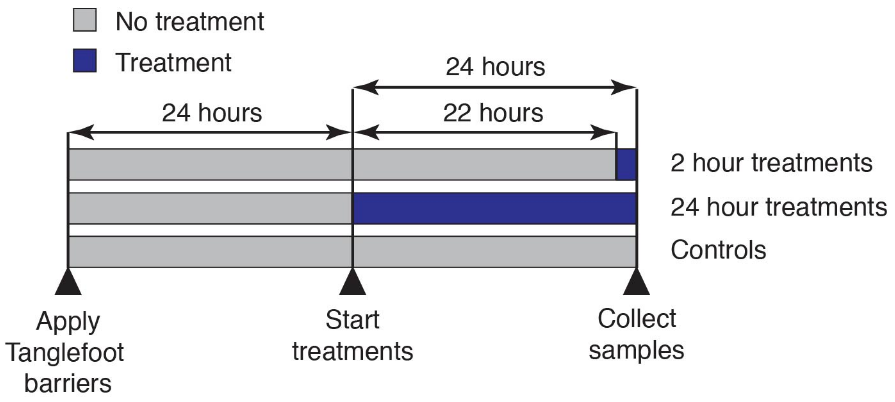

Supplementary Figure 3. Experimental design for collection of RNA for transcriptomic analyses. Tanglefoot barriers were applied to barley and maize leaves 48 hours before the time of tissue collection. At 24 and 2 hours prior to tissue collection, treatments (mite infestation and wounding) were applied to the leaf area bounded by Tanglefoot (Supplementary Figure 1). The control samples received the barriers but none of the treatments. All samples were collected within a 15-minute window to minimize the impact of environmental and circadian effects on gene expression.

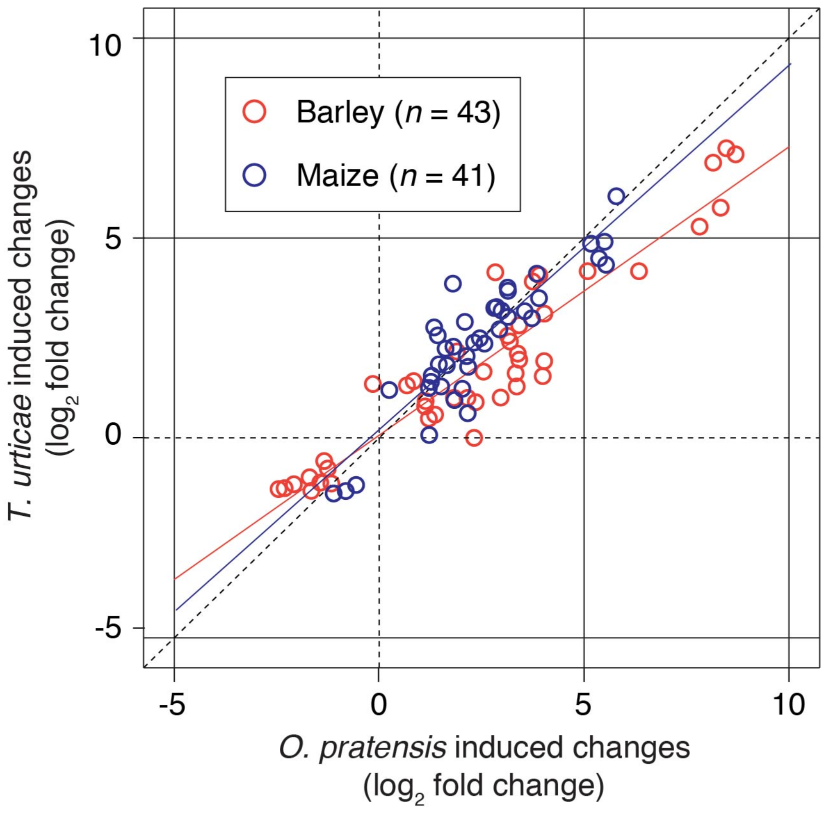

Supplementary Figure 4. Peroxidase genes are differentially expressed in response to spider mite herbivory in barley and maize. Scatter plots of $\log_2$ fold changes for genes differentially expressed in at least one treatment (FDR adjusted *P*-value of 0.01, absolute value $\log_2$ fold change cutoff of 1) with the GO term "peroxidase activity" in barley (orange) and maize (blue) in response to *T. urticae* and *O. pratensis* herbivory at 24 hours after infestation (*n*: sample size).

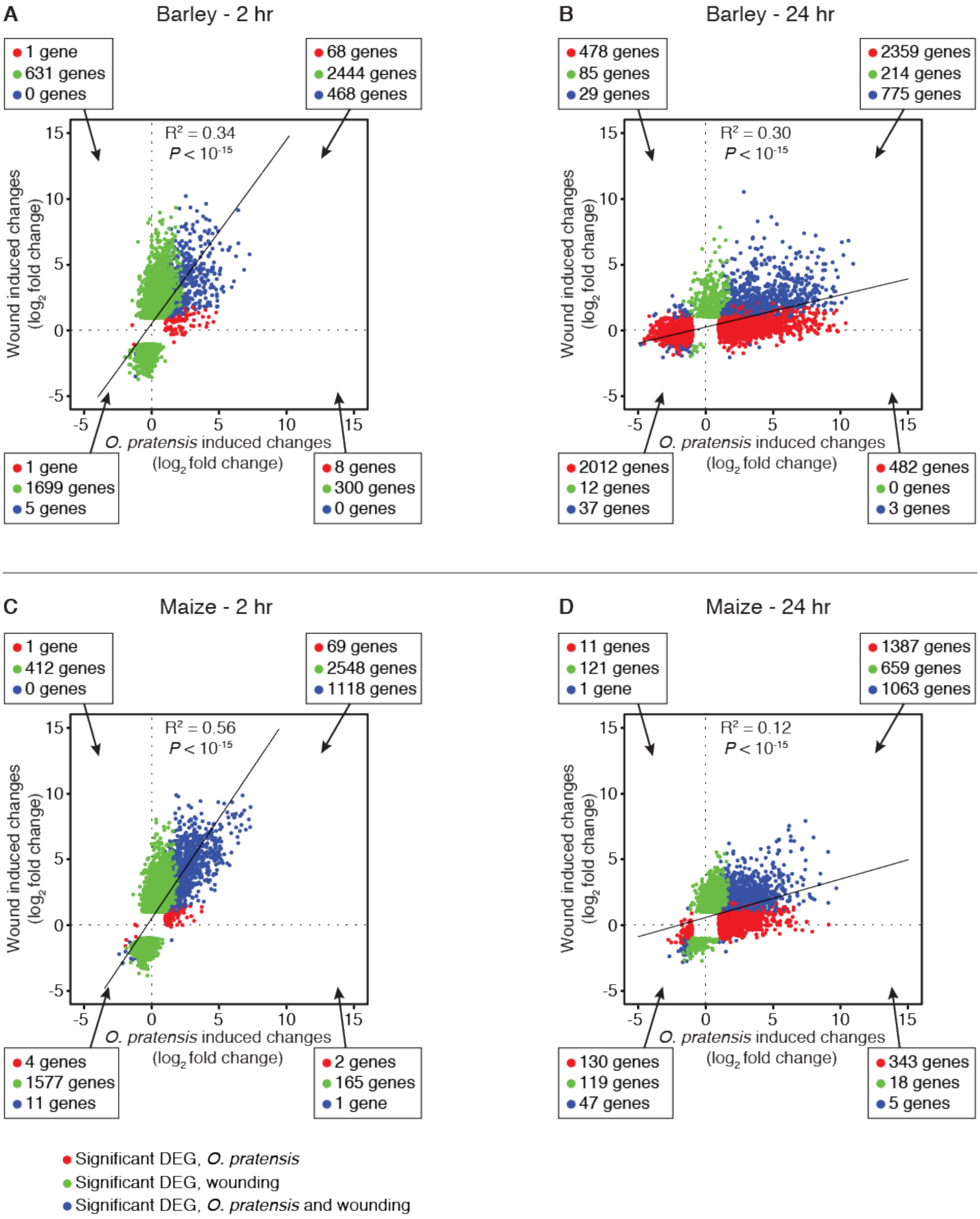

Supplementary Figure 5. Comparison of transcriptional responses to spider mite herbivory and wounding in barley and maize. Scatter plots of log_2 fold changes for genes in barley **(A and B)** and maize **(C and D)** in response to *O. pratensis* herbivory and wounding at two time points (2 hours: **A**

and **C**; 24 hours: **B** and **D**). For inclusion in a given analysis, a gene had to be detected as differentially expressed in response to at least one treatment (FDR adjusted *P*-value of 0.01, absolute value log_2 fold change cutoff of 1). The number of genes in each quadrant is indicated in boxes. Because barley and maize responses to *O. pratensis* and *T. urticae* were extremely similar (**Figure 3**), only scatter plots of expression changes versus wounding for one mite species (*O. pratensis*) are shown; however, as expected, nearly identical results were obtained for the respective analyses between *T. urticae* and wounding (R^2 values were between 0.29 and 0.51, with all P-values $< 10^{-15}$).

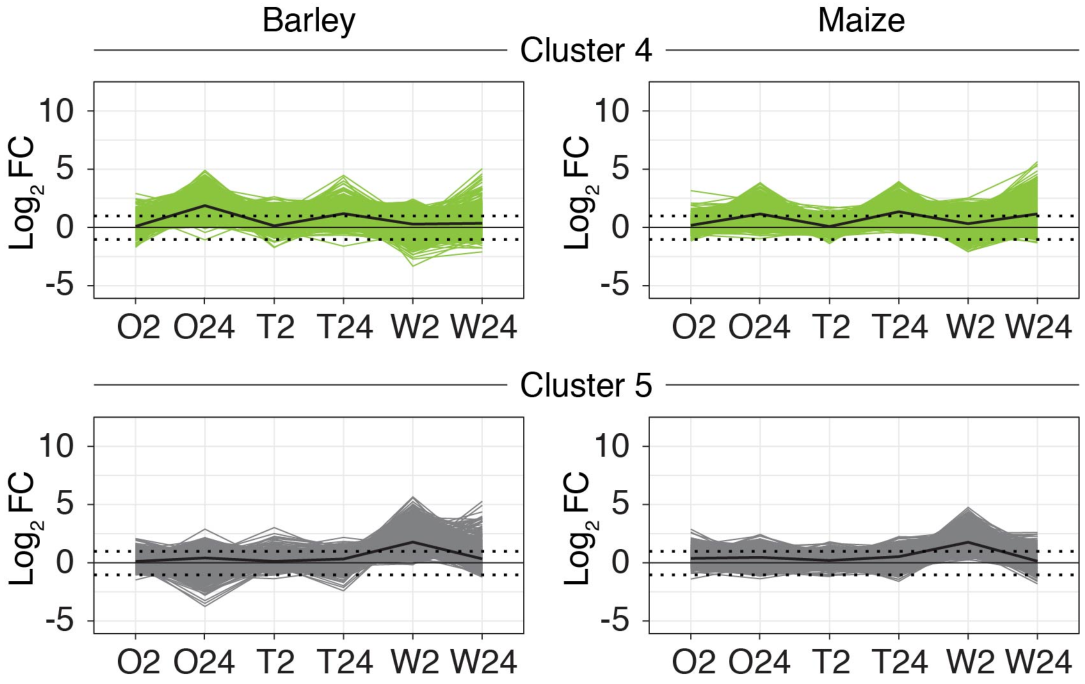

Supplementary Figure 6. Gene expression profiles of barley and maize genes in clusters 4 and 5. The $\log_2$ fold change (FC) expression estimates for individual genes in clusters 4 and 5 are shown with colored lines (compare to **Figure 5A** to match colors with clusters). The average expression changes of all genes in each cluster are shown by black lines. Treatments are O2 and O24: *O. pratensis* herbivory at 2 and 24 hours; T2 and T24: *T. urticae* herbivory at 2 and 24 hours; and W2 and W24: wounding at 2 and 24 hours.

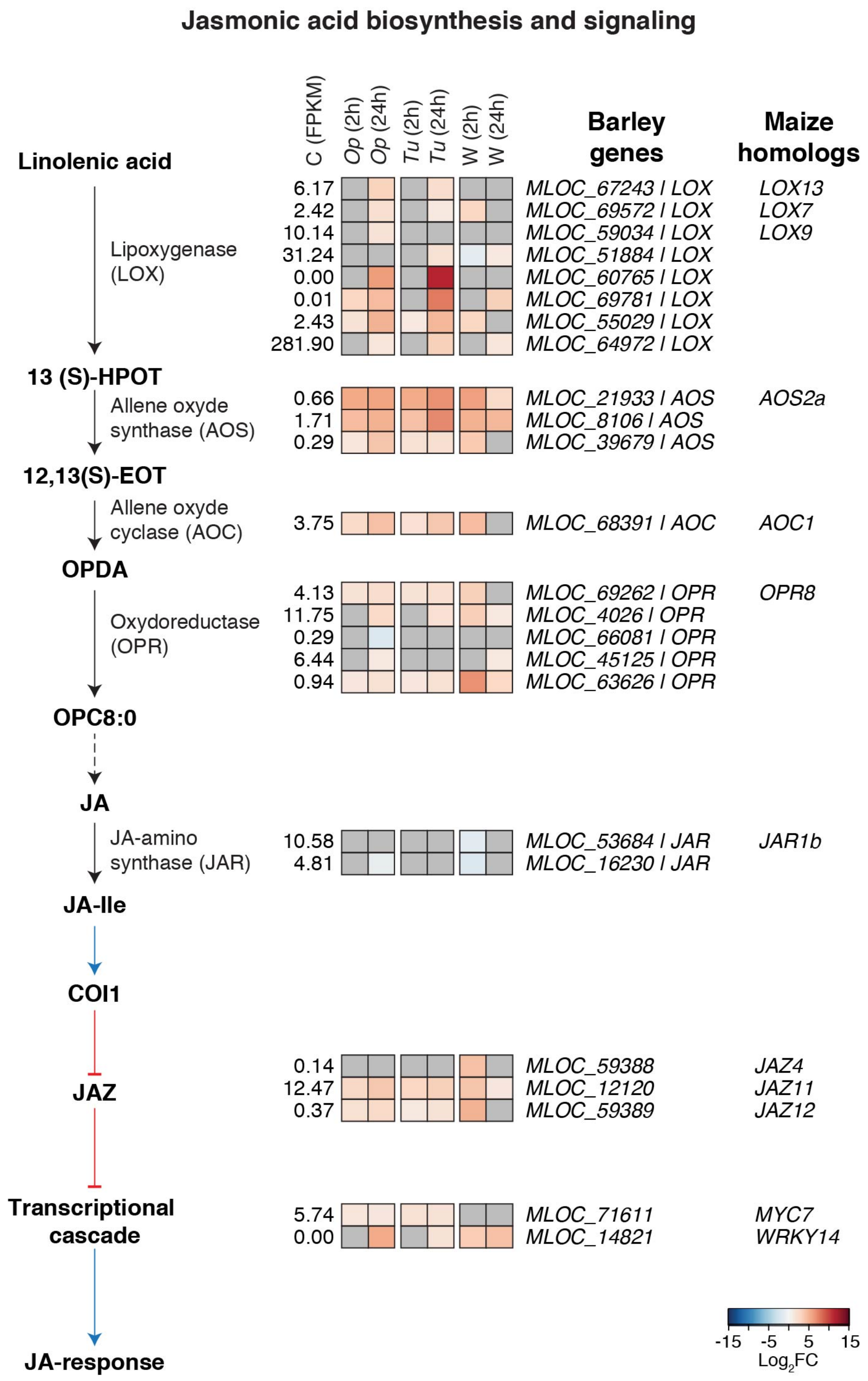

Supplementary Figure 7. Transcriptional changes for JA-associated genes in barley in response to spider mite herbivory and wounding. The putative pathway for JA biosynthesis and signaling is shown at left (see **Figure 6C**). Barley JA synthetic genes are from annotations on the BarleyCyc database (Plant Metabolic Network, PMN12 release, http://www.plantcyc.org), while genes for JA-mediated signaling (e.g., *JAZ* genes*, MYC7,* and *WRKY14*) were inferred by identifying the best reciprocal BLASTP hits of maize JA signaling genes (**Figure 6C**). The average expression level of each gene among control replicates (C) is given in FPKMs. Blue and red colors correspond to $\log_2$

fold changes (FC) for differentially expressed genes (FDR adjusted *P*-value of 0.01, absolute value $\log_2$ fold change cutoff of 1) in given treatments: blue: downregulated; red: upregulated; and gray: not detected as significantly differentially expressed (only genes differentially regulated in at least one contrast are shown). Treatments: *Op*: *O. pratensis*; *Tu*: *T. urticae*; and W: wounding. Time points are 2 and 24 hours (2h and 24h, respectively). Black arrows indicate chemical transformations (solid: one reaction; dashed: multiple reactions) and blue and red arrows indicate signal transduction (activation and inhibition, respectively).

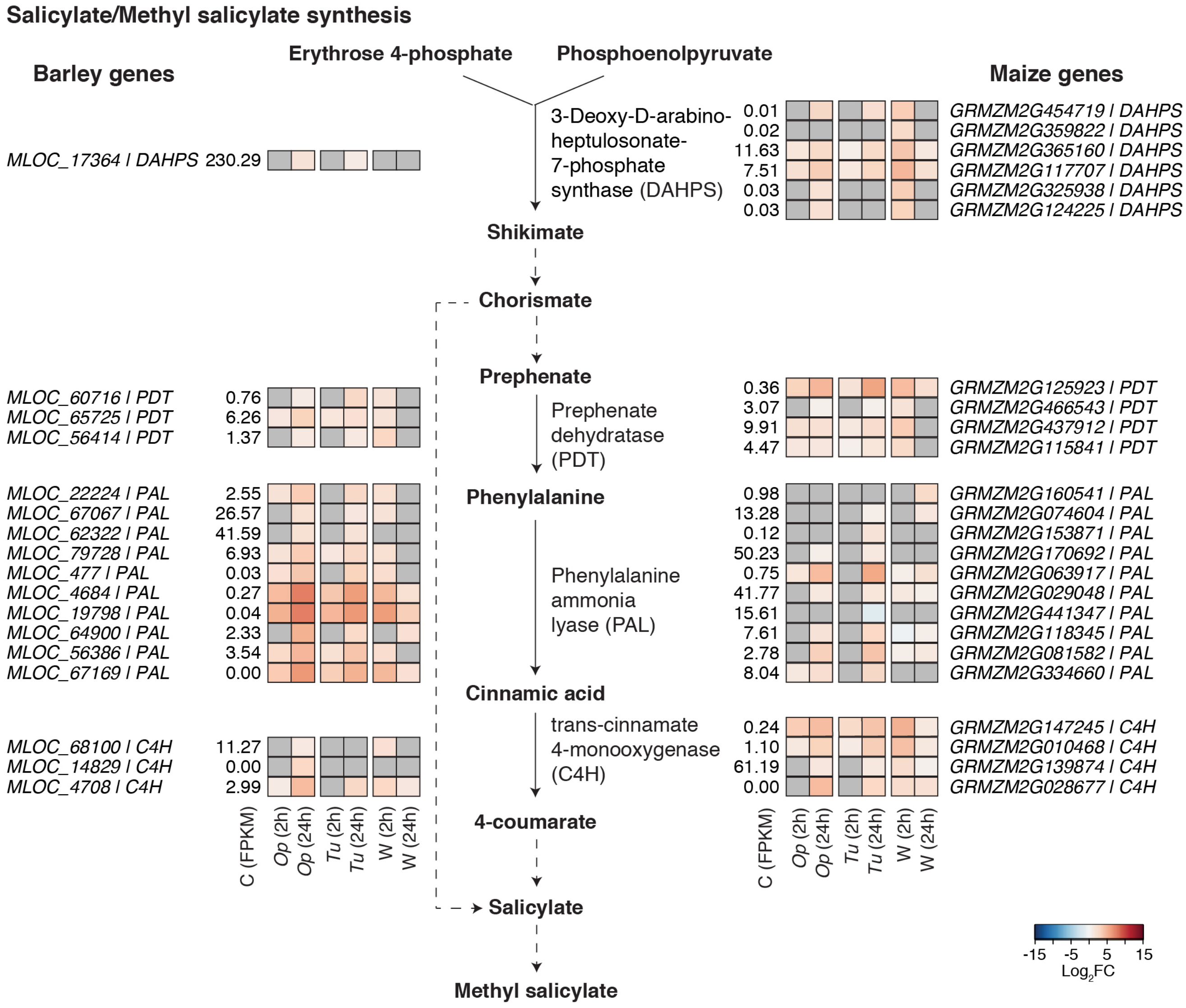

Supplementary Figure 8. Mite herbivory and wounding induce transcriptional changes in barley and maize for putative SA biosynthetic genes. Shown in the middle is the biosynthetic pathway for production of methyl salicylate (Martel et al., 2015; Tzin et al., 2015). Black arrows indicate chemical transformations (solid: one reaction; dashed: multiple reactions). The barley and maize SA synthetic genes were as annotated on the BarleyCyc and CornCyc databases (Plant Metabolic Network, PMN12 release, http://www.plantcyc.org), respectively. Average expression among control (C) replicates for each gene is given in FPKMs. Blue and red colors correspond to $\log_2$ fold changes (FC) for differentially expressed genes (FDR adjusted *P*-value of 0.01, absolute value $\log_2$ fold change cutoff of 1) in given treatments: blue: downregulated; red: upregulated; and gray: not detected as significantly differentially expressed (only genes differentially regulated in at least one contrast are shown). Treatments: *Op*: *O. pratensis*; *Tu*: *T. urticae*; and W: wounding. Time points are 2 and 24 hours (2h and 24h, respectively).

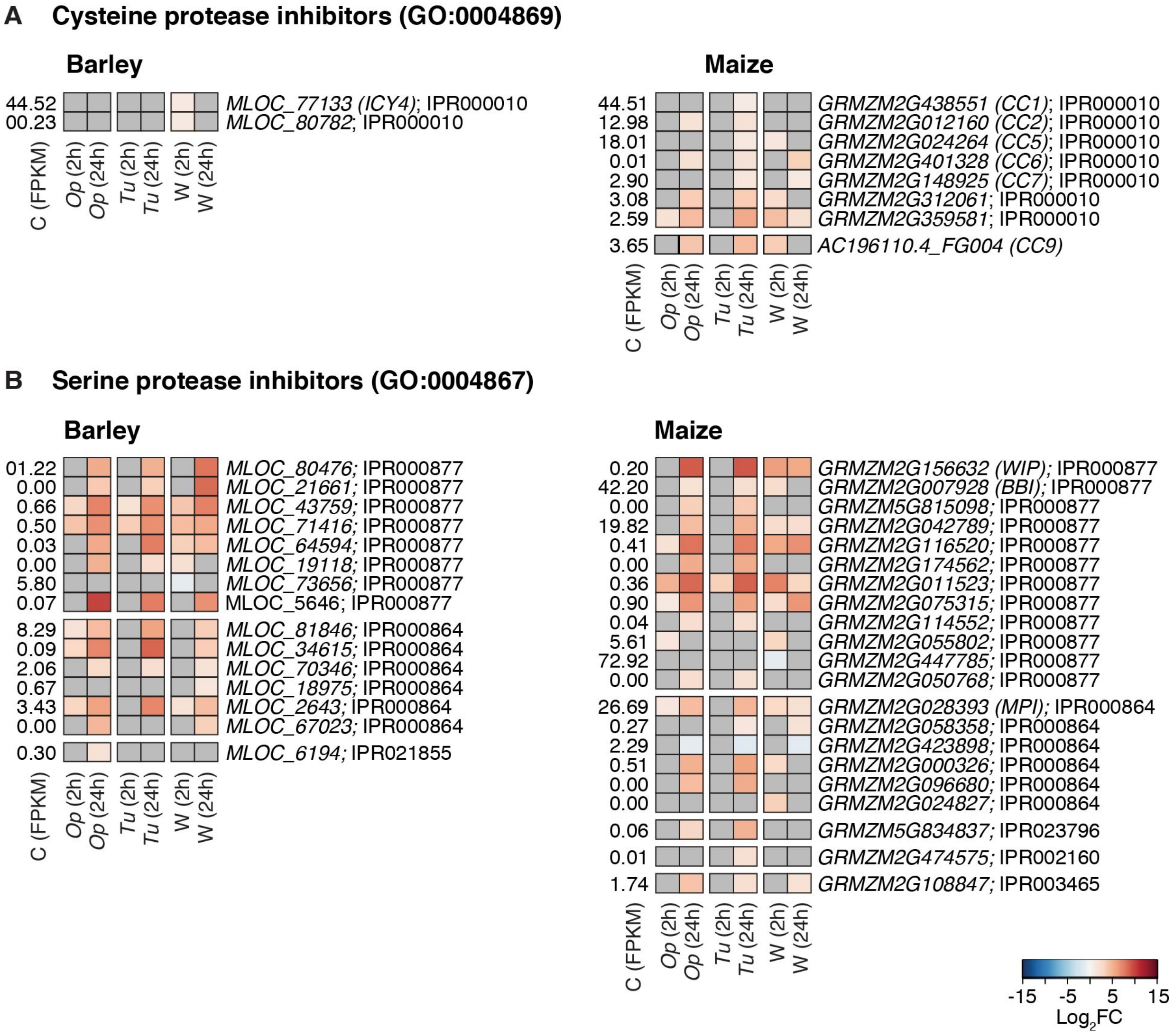

Supplementary Figure 9. Protease inhibitor genes are differentially expressed in response to spider mite herbivory in barley and maize. Heat maps for transcriptional changes for cysteine protease inhibitors (cystatins) **(A)** and serine protease inhibitors **(B)** in response to spider mite herbivory and wounding in barley and maize (left and right, respectively). The gene lists were identified based on the Gene Ontology annotations of the maize and barley genomes as well as previous molecular studies (Martinez et al., 2009; Richter et al., 2016; Rohrmeier and Lehle, 1993; Tamayo et al., 2000). Average expression among control (C) replicates for each gene is given in FPKMs. Blue and red colors correspond to log_2 fold changes (FC) for differentially expressed genes (FDR adjusted *P*-value of 0.01, absolute value log_2 fold change cutoff of 1) in given treatments: blue: downregulated; red: upregulated; and gray: not detected as significantly differentially expressed (only genes differentially regulated in at least one contrast are shown). Treatments: *Op*: *O. pratensis*; *Tu*: *T. urticae*; and W: wounding. Time points are 2 and 24 hours (2h and 24h, respectively).

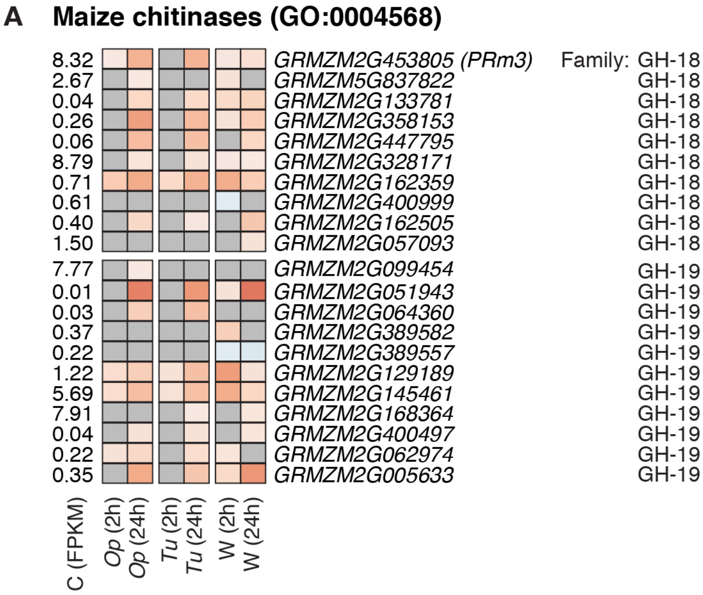

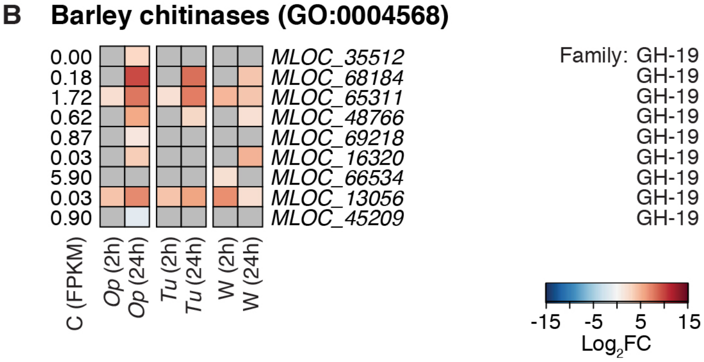

Supplementary Figure 10. Genes encoding chitinases are differentially expressed in response to spider mite herbivory and wounding in barley and maize. Heat maps for transcriptional changes detected in maize **(A)** and barley **(B)** for genes encoding predicted chitinases. The gene lists were identified based on the gene ontology annotations of the maize and barley genomes as well as previous molecular studies (Hawkins et al., 2015; Nasser et al., 1988). Average expression among control (C) replicates for each gene is given in FPKMs. Blue and red colors correspond to log_2 fold changes (FC) for differentially expressed genes (FDR adjusted *P*-value of 0.01, absolute value log_2 fold change cutoff of 1) in given treatments: blue: downregulated; red: upregulated; and gray: not detected as significantly differentially expressed (only genes differentially regulated in at least one contrast are shown). Treatments: *Op*: *O. pratensis*; *Tu*: *T. urticae*; and W: wounding. Time points are 2 and 24 hours (2h and 24h, respectively).

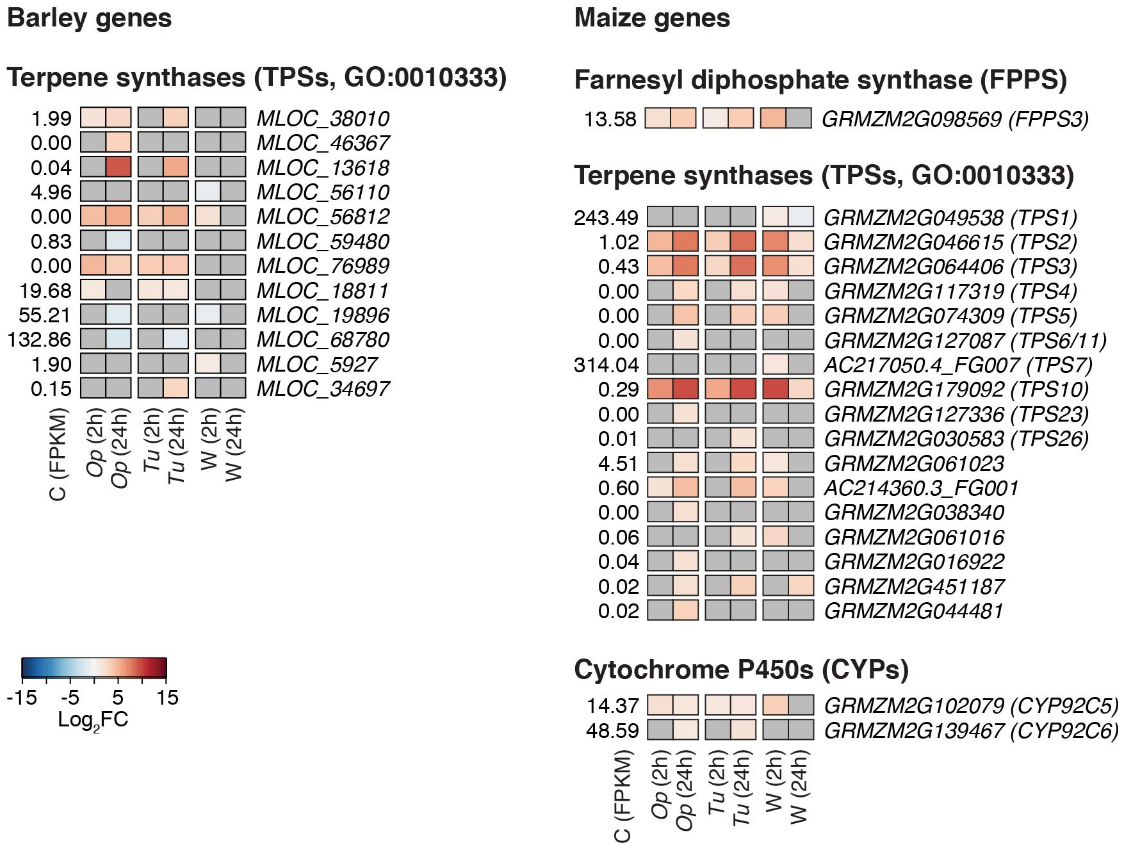

Supplementary Figure 11. Genes encoding proteins for the synthesis of terpenes are differentially expressed in response to spider mite herbivory and wounding in barley and maize. Heat maps for transcriptional changes detected in barley (left) and maize (right) for genes encoding known or putative proteins involved in terpene biosynthesis. The gene lists were identified based on the gene ontology annotations of the maize and barley genomes as well as previous molecular studies (Köllner et al., 2009; Lin et al., 2008; Richter et al., 2016; Schnee, 2002). Average expression among control (C) replicates for each gene is given in FPKMs. Blue and red colors correspond to $\log_2$ fold changes (FC) for differentially expressed genes (FDR adjusted *P*-value of 0.01, absolute value $\log_2$ fold change cutoff of 1) in given treatments: blue: downregulated; red: upregulated; gray: not detected as significantly differentially expressed (only genes differentially regulated in at least one contrast are shown). Treatments: *Op*: *O. pratensis*; *Tu*: *T. urticae*; and W: wounding. Time points are 2 and 24 hours (2h and 24h, respectively).

Supplementary References

Hawkins, L. K., Mylroie, J. E., Oliveira, D. A., Smith, J. S., Ozkan, S., Windham, G. L., et al. (2015). Characterization of the maize chitinase genes and their effect on *Aspergillus flavus* and aflatoxin accumulation resistance. *PLoS One* 10, e0126185. doi:10.1371/journal.pone.0126185.

Köllner, T. G., Gershenzon, J., and Degenhardt, J. (2009). Molecular and biochemical evolution of maize terpene synthase 10, an enzyme of indirect defense. *Phytochemistry* 70, 1139–1145. doi:10.1016/j.phytochem.2009.06.011.

Lin, C., Shen, B., Xu, Z., Kollner, T. G., Degenhardt, J., and Dooner, H. K. (2008). Characterization of the monoterpene synthase gene *tps26*, the ortholog of a gene induced by insect herbivory in maize. *Plant Physiol.* 146, 940–951. doi:10.1104/pp.107.109553.

Martel, C., Zhurov, V., Navarro, M., Martinez, M., Cazaux, M., Auger, P., et al. (2015). Tomato whole genome transcriptional response to *Tetranychus urticae* identifies divergence of spider mite-induced responses between tomato and *Arabidopsis*. *Mol. Plant. Microbe Interact.* 28, 343–361. doi:10.1094/MPMI-09-14-0291-FI.

Martinez, M., Cambra, I., Carrillo, L., Diaz-Mendoza, M., and Diaz, I. (2009). Characterization of the entire cystatin gene family in barley and their target cathepsin l-like cysteine-proteases, partners in the hordein mobilization during seed germination. *Plant Physiol.* 151, 1531–1545. doi:10.1104/pp.109.146019.

Nasser, W., de Tapia, M., Kauffmann, S., Montasser-Kouhsari, S., and Burkard, G. (1988). Identification and characterization of maize pathogenesis-related proteins. Four maize PR proteins are chitinases. *Plant Mol. Biol.* 11, 529–538. doi:10.1007/BF00039033.

Richter, A., Schaff, C., Zhang, Z., Lipka, A. E., Tian, F., Köllner, T. G., et al. (2016). Characterization of biosynthetic pathways for the production of the volatile homoterpenes DMNT and TMTT in *Zea mays*. *Plant Cell* 28, 2651–2665. doi:10.1105/tpc.15.00919.

Rohrmeier, T., and Lehle, L. (1993). WIP1, a wound-inducible gene from maize with homology to Bowman-Birk proteinase inhibitors. *Plant Mol. Biol.* 22, 783–792. doi:10.1007/BF00027365.

Schnee, C. (2002). The maize gene *terpene synthase 1* encodes a sesquiterpene synthase catalyzing the formation of (E)-beta-farnesene, (E)-nerolidol, and (E,E)-farnesol after herbivore damage. *Plant Physiol.* 130, 2049–2060. doi:10.1104/pp.008326.

Tamayo, M. C., Rufat, M., Bravo, J. M., and San Segundo, B. (2000). Accumulation of a maize proteinase inhibitor in response to wounding and insect feeding, and characterization of its activity toward digestive proteinases of *Spodoptera littoralis* larvae. *Planta* 211, 62–71. doi:10.1007/s004250000258.

Tzin, V., Fernandez-Pozo, N., Richter, A., Schmelz, E. A., Schoettner, M., Schäfer, M., et al. (2015). Dynamic maize responses to aphid feeding are revealed by a time series of transcriptomic and metabolomic assays. *Plant Physiol.*, pp.01039.2015. doi:10.1104/pp.15.01039.
